# Graphical pangenomics-enabled characterization of structural variant impact on gene expression in *Brassica napus*

**DOI:** 10.1007/s00122-025-04867-2

**Published:** 2025-04-03

**Authors:** Gözde Yildiz, Silvia F. Zanini, Sven Weber, Venkataramana Kopalli, Tobias Kox, Amine Abbadi, Rod J. Snowdon, Agnieszka A. Golicz

**Affiliations:** 1 Department of Agrobioinformatics, IFZ Research Center for Biosystems, Land Use and Nutrition, Justus Liebig University, Heinrich Buff Ring 26-32, 35392 Giessen, Germany; 2 Department of Plant Breeding, IFZ Research Center for Biosystems, Land Use and Nutrition, Justus Liebig University, Heinrich Buff Ring 26-32, 35392 Giessen, Germany; 3 NPZ Innovation GmbH, Hohenlieth-Hof, 24363 Holtsee, Germany

## Abstract

**Key message:**

Pangenome graphs enable population-scale genotyping and improve expression analysis, revealing that structural variations (SVs), particularly transposable elements (TEs), significantly contribute to gene expression variation in winter oilseed rape.

**Abstract:**

Structural variations (SVs) impact important traits, from yield to flowering behaviour and stress responses. Pangenome graphs capture population-level diversity, including SVs, within a single data structure and provide a robust framework for downstream applications. They have the potential to serve as unbiased references for SV genotyping, pan-transcriptomic analyses, and association studies, offering significant advantages over single reference genomes. However, their full potential for expression quantitative trait locus (eQTL) analysis is yet to be explored. We combined long and short-read whole genome sequencing data with expression profiling of *Brassica napus* (oilseed rape) to assess the impact of SVs on gene expression regulation and explored the utility of pangenome graphs for eQTL analysis. Over 90,000 SVs were discovered from 57 long-read datasets. Pangenome graph as reference was evaluated and used for SV genotyping with short reads and transcript expression quantification. Using SVs genotyped from the graph and 100 expression datasets, we identified 267 gene proximal (cis) SV-eQTLs. Over 70% of eQTL-SVs had similarity to transposable elements (TEs), especially Helitrons. The highest proportion of cis-eQTL-SVs were found in promoter regions. About a third of transcripts whose expression was associated with SVs, had no associated SNPs, suggesting that including SVs allows capturing of relationship which would be missed in SNP-only analyses. This study demonstrated that pangenome graphs provide a unifying framework for eQTL analysis by allowing population-scale SV genotyping and gene expression quantification. We also showed that SVs make an appreciable contribution to gene expression variation in winter oilseed rape.

**Supplementary Information:**

The online version contains supplementary material available at 10.1007/s00122-025-04867-2.

## Introduction

Structural variations (SVs) are genomic alterations over 50 bp in length, with insertions and deletions representing the most common forms (Alonge et al. 2020; Yildiz et al. 2023). SVs are prevalent in the complex genomes of major crops including wheat (Walkowiak et al. 2020), barley (Jayakodi et al. 2020), and oilseed rape (Chawla et al. 2021). They are associated with key traits such as yield and flowering time in oilseed rape (Song et al. 2020), fruit flavour in tomato (Li et al. 2023), and quality traits in cotton (Jin et al. 2023). SVs can impact gene function by altering protein-coding sequences, splicing patterns, gene expression levels, or any combination thereof (Chiang et al. 2017; Zanini et al. 2022). Expression quantitative trait loci (eQTL) analysis maps associations between genomic variation and gene expression. Results from eQTL studies are often used in conjunction with classical QTL mapping or genome-wide association studies (GWAS) to pinpoint causal or candidate genes (Druka et al. 2010). They can however also be used to help understand the regulatory architecture of gene expression and complex phenotypic traits. The most common variants used in eQTL studies are single nucleotide polymorphisms (SNPs), however, due to increasing capacity for population-scale SV discovery (Alonge et al. 2020; Chawla et al. 2021; Zhang et al. 2022), the impact of SVs on genome-wide expression patterns can now also be investigated in large scale eQTL analyses (Leonard et al. 2024).

Recently, pangenome graphs have emerged as a robust framework for genomic data analysis, capturing species-wide genomic diversity within a single data structure (Yildiz et al. 2022; Zanini et al. 2022; Hu et al. 2024). The main methods for constructing plant pangenomes are de novo assembly and comparison, reference genome-based iterative assembly, and graph-based pangenome approach (Hu et al. 2024). In the de novo assembly method, individual genomes are assembled from scratch to identify shared and unique regions. Subsequent analyses commonly focus on comparing gene annotations across genomes, emphasizing the species' pangene set. The iterative mapping and assembly method starts by aligning reads to an existing reference genome. Reads that don’t align are then assembled, and the resulting annotated contigs are integrated into a linear pangenome reference, allowing for the representation of all sequences but compromising on their positional relationships (Golicz et al. 2016; Jain and Garg 2020). The third approach, graph-based pangenomes, represents all genomic sequences and variants as nodes and edges, offering major advantages over reference-based genomes, including:(1) reduced redundancy, by integrating multiple genome sequences into a single graph structure that preserves linear proximity of nodes, even in the presence of complex rearrangements; (2) improved read mapping accuracy and variant detection, by capturing large SVs and unique alleles that may not be represented in single reference genomes or linear pangenomes, and (3) provides a more comprehensive and unbiased reference for association studies (Edwards and Batley 2022).

A necessary prerequisite of association studies is that genomic variations across large populations need to be genotyped accurately and rapidly (Wang et al. 2018; Fuentes et al. 2019). Traditional genotyping methods align short reads to a single reference genome (Alkan et al. 2011; DePristo et al. 2011). However, read alignment errors caused by single reference bias result in inaccurate genotypes, especially for alternative alleles (Cameron et al. 2019). Therefore, graph-based SV genotyping methods using short reads emerged as a powerful alternative (Liu et al. 2020; Lemay et al. 2022; Li et al. 2022; Leonard et al. 2024). Graph-based genotyping algorithms use either read alignment or k-mer matching against the variation/sequence graphs to genotype variants using short reads (Chen et al. 2019; Hickey et al. 2020). However, these methods still have some limitations, being mainly optimized for human genomes, with only limited benchmarking on crop genomes (Lemay et al. 2022; Du et al. 2024). Additionally, crop genomes can present unique challenges for SV genotyping due to their complexity, including differences in genome size, high repeat content, heterozygosity, and polyploidy. Beyond its utility for SV genotyping, pangenome graphs can also be utilized for pan-transcriptomic analyses (Sibbesen et al. 2023), where genomic variation is accounted for during mRNA-Seq read mapping and subsequent quantification.

In this study, we combined long-read Oxford Nanopore (ONT) and short-read Illumina genome sequencing data with mRNA-Seq data from young leaves of *B. napus* (oilseed rape) to assess the impact of SVs on gene expression regulation and explore the utility of pangenome graphs for eQTL analysis in plants. We assessed the effectiveness of graph-based SV genotyping using state-of-the-art approaches and further tested the utility of pangenome graphs for transcript expression quantification. We found that insertions, deletions and especially transposable elements (TEs) contribute to gene expression diversity and that some of the associations could not be detected using only SNPs, highlighting the importance of integrating SVs in association studies to understand the impact of different types of mutations on crop traits.

## Materials and methods

### Material selection

A total of 100 genetically diverse, elite inbred winter oilseed rape breeding lines from the commercial breeding programme of Norddeutsche Pflanzenzucht HG Lembke (NPZ KG, Hohenlieth, Germany) were used in the study. All 100 lines were used for short-read sequencing. Based on genetic diversity analysis using genome-wide SNPs called from the short-read data, a subset of 57 lines representing the total genetic diversity of the full collection was selected for long-read sequencing. Single plants from each inbred line were harvested for the short and long-read sequencing, respectively.

### Short-read genomic and RNA-Seq sample preparation and sequencing

Plants were grown in a climate-controlled growth chamber with 16-h day (16 °C) and 8-h night (12 °C). Leaf samples were harvested simultaneously for all genotypes after 30 days at the 5–6 leaf stage, immediately shock-frozen in liquid nitrogen, and stored at − 80 °C until DNA/RNA extraction. Leaf material was then ground to a fine powder in liquid nitrogen and separated into aliquots for DNA and RNA extraction. Total genomic DNA was extracted from each sample using the CTAB extraction method of Doyle (1990). Total RNA was extracted using the RNeasy Mini Kit (Qiagen, Hilden, Germany) and treated using RNase-free DNase (Qiagen, Hilden, Germany) to remove DNA. Quantity and quality of RNA samples were checked using a Fragment Analyzer Automated Capillary Electrophoresis system (Advanced Analytical, Heidelberg, Germany). Equimolar RNA/DNA samples were shipped on dry ice to BGI Tech Solutions (Hong Kong, China) for library preparation and sequencing. Whole-genome DNA sequencing was performed with 150nt paired-end reads on the Illumina Hiseq XTen platform. RNA-Seq was performed on the Illumina HiSeq 4000 platform with 100nt paired-end sequencing.

### Long-read genomic sample preparation and sequencing

Plants were grown in the same conditions as for short-read sequencing, leaves were harvested from plants at the 4–6 leaf stage, flash frozen, and ground to a fine powder using a mortar and pestle. High-molecular-weight DNA was isolated and sequenced using a modified protocol from Chawla et al (2021). Briefly, 11 mL of pre-heated lysis buffer (1% w/v PVP40, 1% w/v PVP10, 500 mm NaCl, 100 mm TRIS pH8, 50 mm EDTA, 1.25% w/v SDS, 1% (w/v) Na_2_S_2_O_5_, 5 mm C_4_H_10_O_2_S_2_, 1% v/v Triton X-100) were added to 1.2–1.5 g of tissue and incubated for 30 min at 37 °C in a rotator. 11 μl RnaseCocktail (ThermoFisher, ref AM2288) were added and the lysate was incubated in a rotator at 37 °C for 20 min. 110 μl of ProteinaseK (ThermoFisher, ref QS0511) were then added and samples were incubated in a rotator at 37 °C for a further 20 min. 4 mL of 5 M potassium acetate were added to the cooled-down lysate, mixed by inversion 20 times, incubated for 10 min on ice, and pelleted by centrifugation at 4 °C, 4250 g for 10 min.

Finally, magnetic beads were used to recover the HMW-DNA, washed twice with 70% ethanol, and incubated with TE buffer for 10 min at 37 °C to release the DNA from the beads into the buffer. 1 to 3 ug of DNA were used for library preparation with the ligation sequencing kit SQK-LSK109, according to the manufacturer’s recommendations, and loaded onto an Oxford Nanopore MinION flow cell for sequencing.

### SV calling from long reads

In our previous work, we established optimal combinations of alignment and SV calling methods for low to medium sequencing depths (Yildiz et al. 2023). Long-read datasets (*n*: 57) were aligned against reference *B. napus* genome (Express 617 v1) (Lee et al. 2020) using minimap2 v2.24-r1122 (Li 2018), followed by sorting and indexing of the aligned reads with samtools v1.9 (Li et al. 2009). Subsequently, cuteSV v1.0.13 (Jiang et al. 2020) was used to detect SVs with varying coverages; 5x (13 lines), 10x (25 lines), and 20x (19 lines) designated as –min_support values 3, 5, and 8, respectively. SVs genotyping option (–genotype) was enabled and calls across samples were merged using Jasmine v1.0.2 (Kirsche et al. 2023). Merged SVs were re-genotyped with cuteSV (-lvcf). Variants were further processed to only retain insertions and deletions with genotype missing call rate: < 5%, heterozygous genotype call rate: < 5% and remove variants > 20 kb. This approach was based on our earlier findings (Yildiz et al. 2023), which highlighted insertions and deletions as the most prevalent variant types in *B. napus* and were associated with lowest detection errors. Additionally, heterozygous SVs were excluded from analysis, due to potentially erroneous genotype calls in the highly inbred lines used in the analysis.

### SNP calling from short reads

Short reads (*n*: 57) were aligned to the reference genome (Express 617 v1) using bwa-mem2 v2.2.1 (Vasimuddin et al. 2019). SNP calling was performed with bcftools v1.15.1 mpileup –skip-indels –min-MQ 10 (minimum read mapping quality) and bcftools call -mv -Ov –ploidy 2 (Danecek et al. 2021). SNPs were filtered using similar criteria as for SVs calling, retaining variants with genotype missing call rate: < 5%, heterozygous genotype call rate: < 5% and minor allele frequency: > 5%.

### Graph-based SV genotyping

We performed graph-based SV genotyping using Paragraph v.2.4a (Chen et al. 2019), vg toolkit: v1.43.0 Giraffe/vg (Hickey et al. 2020; Sirén et al. 2021), and v1.1.8 Ensemble Variant Genotyper (EVG) (Du et al. 2024) on 57 short-read datasets. SV genotyping tools included in the study were selected using several criteria: (1) all of them perform graph-based SV genotyping, (2) Giraffe/vg appears to be the most popular genotyper in literature to date (Liu et al. 2020; Sirén et al. 2021; Li et al. 2023), (3) Paragraph is the best performing genotyper based on benchmarking in soybean (Lemay et al. 2022), (4) EVG combines multiple graph-based SV genotyping algorithms. For Giraffe/vg genotyping short reads were aligned to the pangenome graph using vg giraffe (Sirén et al. 2021). SVs from long reads (*n*: 57) were used in vg autoindex v1.43.0 –workflow giraffe. SVs were genotyped using vg pack and vg call with default parameters (read support with -Q 5, ignore mapping and base quality below 5, -s 5, ignore first and last 5 bp from each read). For Paragraph, SVs from long reads (*n*: 57) were provided along with Express 617 v1 reference genome and genotyping was done using default parameters. For EVG, graph SVs (*n*: 57) and Express 617 v1 reference genome were provided, and genotyping was performed with default parameters.

### F1-score calculation

We calculated F1-scores for each variant using SV genotypes obtained from different short-read genotypers and long-read SV genotypes used as the truth set.

$$F1=\frac{2*(\text{precision}*\text{recall})}{\text{precision}+\text{recall}}= \frac{2\text{TP}}{2\text{TP}+\text{FP}+\text{FN}}$$

F1-scores were calculated both from the perspective of alternative (ALT: non-reference) and reference (REF) alleles as it can lead to somewhat different results. For example, for the 57 lines, a variant which in the truth set had alternative allele call in two lines, but was genotyped from short reads as alternative allele in 3 lines, will have an F1-score of 0.8 ((2*TP:2)/(2*TP:2 + FP:1 + FN:0)) for the ALT allele, but F1-score of 0.99 ((2*TP:54)/(2*TP:54 + FP:0 + FN:1)) for the REF allele. Heterozygous calls were treated as missing and not included in the calculation, and variants which had > 20% missing rate were designated F1-score of zero.

### Comparison of SV with low and high F1-scores

Properties of SVs with low and high F1-scores were compared with respect to length, location, copy number and initial SV calling accuracy. SVs were considered gene proximal if they were within 1 kb of protein-coding genes. Sequences of all variants were extracted and used as query in a BLAST search (with -blastn -evalue 1e-5 -outfmt 6) against Express 617 v1 genome to see if the sequences corresponding to variants with low F1-score have a higher genome-wide copy number.

### Transcript expression quantification from RNA-Seq reads

A pangenome graph was built using vg v1.4.30 (Garrison et al. 2018) autoindex, based on the Express 617 v1 reference genome sequence and using SNPs and SVs which passed the quality control filtering steps described above. RNA-Seq reads were mapped to the graph using vg mpmap. The mappings were passed to rpvg for quantification. For each sample, rpvg outputs quantification results along with haplotype probabilities above a certain threshold. Per-sample results were filtered to retain only haplotypes with the highest probability for each gene. Further, only genes for which the haplotype could be assigned in all samples were retained. Transcripts per million (TPM) values were extracted directly from the rpvg output. Kallisto v0.44.0 (Bray et al. 2016) was used for quantification using transcripts extracted from the Express 617 v1 assembly. TPM values were extracted directly from Kallisto outputs. Transcripts quantified with Kallisto, which could not be assigned a haplotype by rpvg for all samples, were removed prior to comparisons. Pearson and Spearman correlations were calculated for each gene across 50 samples. Transcripts with Pearson correlation below 0.75 were tested for over-representation of SNPs and SVs using a permutation test implemented in regioneR v1.26.1 (Gel et al. 2016). All transcripts quantified by rpvg in all samples were used as a universe for resampling in 100 iterations.

### Simulation of RNA-Seq reads from pangenome graph

RNA-Seq reads were simulated from the pangenome graph using a previously described approach (Sibbesen et al. 2023). In short, haplotype-specific transcripts for one of the samples and their corresponding sequences were extracted from the graph. These served as a reference for gene expression quantification using RNA-Seq data for the same sample with RSEM v1.3.3 (Li and Dewey 2011), generating expression levels from paired-end RNA sequencing reads. These were in turn provided to vg sim v1.57 to simulate corresponding expression levels.

### eQTL identification

Gene expression quantification was performed using rpvg. Only transcripts with mean TPM ≥ 0.1 and expression ≥ 1 TPM in at least two samples were retained for eQTL analysis. The expression matrix was transformed using inverse normal transformation. Five top principal components (PCs) identified from SNP data and top components identified from expression data using the Elbow method of PCAforQTL v0.1.0 (Zhou et al. 2022) were used as covariates in matrixEQTL v2.3 (Shabalin 2012). eQTL analysis was performed jointly for SNPs and SVs. For comparison between short (*n* = 100, *n* = 57) and long (*n* = 57) reads, prior to eQTL analysis variants were further filtered to remove variants with minor allele frequency < 10%, ensuring that minor allele is found in at least five samples. For the final eQTL analysis (*n* = 100), a more relaxed MAF threshold of 5% (also ensuring that minor allele was found in at least five samples) was applied and lead variants were identified by lowest *p*-value. When SNPs and SVs had equal lowest *p*-value, both were retained.

cis-eQTL variants were defined as + / − 3,000 bp from the target gene body, encompassing promoter (3 kb upstream), transcription start site (TSS), exons, introns and 3 kb downstream from the transcription termination site (TTS).

Throughout the manuscript text, “eQTL”, “SV-eQTL” and “SNP-eQTL” refer to a variant-locus pair, while “eQTL-SNP” and “eQTL-SV” refer to the variant only.

### Transposable element annotation

Transposable element library for the Express 617 genome assembly was generated using EDTA v2.0.1 (Ou et al. 2019). Transposable elements were annotated using RepeatMasker v4.1.2 (Smit et al. 2015). Sequences of insertions and deletions were extracted and compared against the TE library using BLASTn v2.13.0 (Camacho et al. 2009) (e-value cutoff 1e-5). Top BLAST matches were used to assign SV sequences to TE families (Lemay et al. 2022). EDTA/ classification of Helitrons was further confirmed comparing it to the output of a Helitron-specific annotation tool, Heliano (v 1.2.1) (Li et al. 2024). Positional overlaps were computed with bedtools intersect (v2.30, -f 0.5). Above 90% of EDTA families classified as Helitron were confirmed by positional overlap with Heliano annotations, therefore EDTA annotation was used for downstream analysis.

### Arabidopsis homologue identification and GO term annotation

Homologue identification was performed using a previously developed method (Golicz et al. 2021; Sessa et al. 2023). Briefly, protein sequences of *B. napus* transcripts were compared against *Arabidopsis thaliana* proteome database using BLASTp v2.13.0 (e-value cutoff 1e-5). Top BLAST matches of *B. napus* transcripts were identified as homologues. GO annotation was performed by transferring TAIR GO annotation of *A. thaliana* to the *B. napus* homologous genes.

## Results

### Winter oilseed rape harbours extensive structural variation

**Fig. 1:**
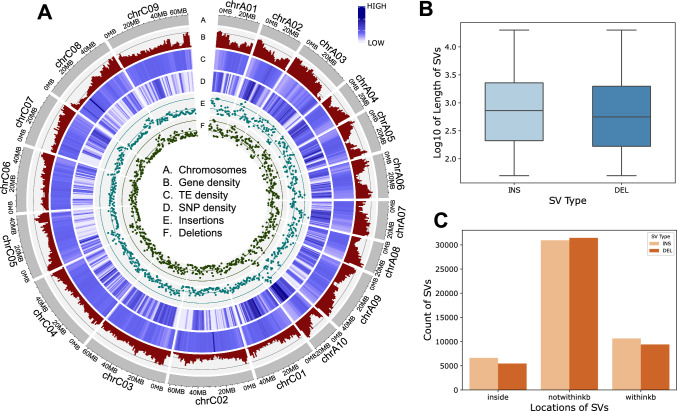
**A** Distribution of various genomic features of the *B. napus* genome, Chromosomes (**A**), Gene density (**B**), TE density (**C**), SNP density (**D**), Insertions (**E**) and Deletions (**F**). Densities were calculated using 1 Mb window size. **B** Distribution of insertion (INS) and deletion (DEL) lengths. (**C**) Distribution of locations of insertion and deletion SVs relative to annotated genes (inside, not within one kb distance and within one kb distance from genes)

Structural variant (SV) discovery was performed using Oxford Nanopore (ONT) long-read sequencing data for 57 lines of German winter oilseed rape. The average coverage for long-read data was 16.5x. Following removal of variants with excessive heterozygous genotype calls (unexpected in highly inbred material and indicative of SV calling/genotyping errors) and excessive genotype missing rate we discovered total of 94,824 structural variations, including 48,396 insertions (INS) and 46,428 deletions (DEL) (Fig. 1A). Deletions averaged 1,745 base pairs in length (median: 561.0), while insertions averaged 1,724 base pairs (median: 727.0). These resulted in a total of 164 Mb of SV space, of which 83 Mb consisted in insertions and as such were not represented in a single reference genome. The length distribution of SVs is represented in Fig. 1B. Regarding their genomic locations, 12.80% of SVs were found within genes (inside), 21.22% of SVs within 1 kb of genes (withinkb), and the majority 65.98% of SVs, in intergenic regions (notwithinkb) (Fig. 1C). Insertions and deletions had similar distribution across genic and non-genic regions (Fig. 1C).

### Graph-based approach allows population-scale SV genotyping

**Fig. 2:**
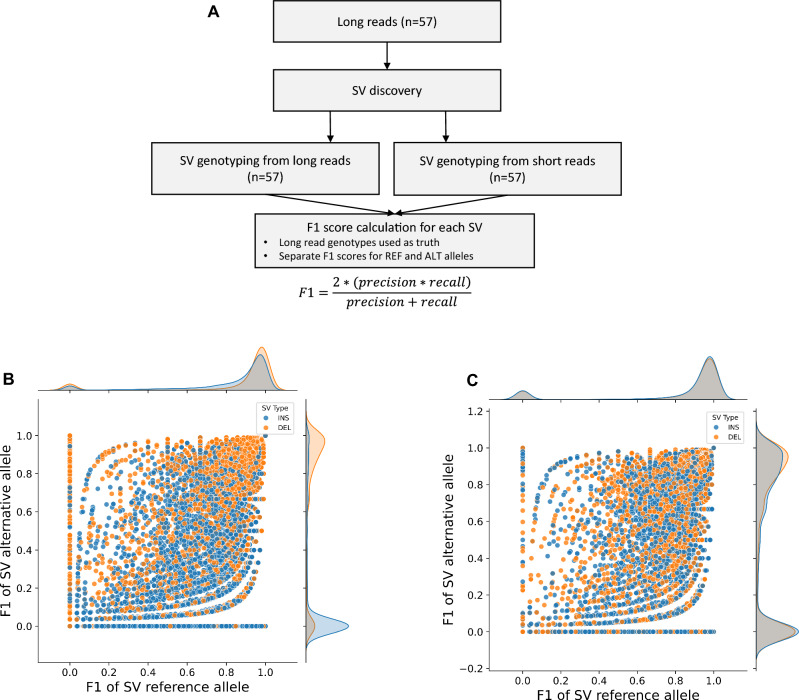
**A** Procedure for F1-score calculation for genotypes obtained from short reads with Giraffe/vg and Paragraph. **B** F1-scores from Giraffe/vg graph-based genotyping for reference (REF) and alternative (ALT) alleles. **C** F1-scores from Paragraph graph-based genotyping for reference (REF) and alternative (ALT) alleles. F1-scores ≤ 0.2 for either alternate or reference alleles are indicative of poor genotyping outcomes, SVs with F1 ≥ 0.8 for both alternate and reference alleles are considered correctly genotyped

SV genotyping from population-level datasets, for example using short Illumina WGS data, is a prerequisite for association analyses. Graph-based SV genotyping from short reads has been shown to be the leading approach, however results from different pipelines vary (Chen et al. 2019; Hickey et al. 2020; Du et al. 2024). We tested three graph-based SV genotyping methods, including Paragraph (Chen et al. 2019), Giraffe/vg (Hickey et al. 2020), and EVG (Du et al. 2024). SVs discovered and genotyped from long reads across 57 samples were used a truth set (Fig. 2A). We then genotyped SVs using short reads derived from the same 57 samples (average coverage 12x). Because matched samples sequenced using ONT and Illumina technology were available, F1-scores could be calculated for each SV individually, checking long- and short-read genotype concordance across samples.

We observed a bimodal distribution of F1-scores (Fig. 2B, C), either close to one (well genotyped, ≥ 0.8) or close to zero (poorly genotyped, ≤ 0.2), in both Paragraph and Giraffe/vg results. However, Giraffe/vg had poorer performance compared to Paragraph, especially for genotyping insertion alternative alleles (Fig. 2B). Giraffe/vg correctly genotyped 34.14% of SVs (SVs with REF allele F1 ≥ 0.8 AND ALT allele F1 ≥ 0.8), while 45.27% of SVs (SVs with REF F1 ≤ 0.2 OR ALT F1 ≤ 0.2) were incorrectly genotyped (Fig. 2B). Paragraph correctly genotyped 43.48% of SVs (SVs with REF allele F1 ≥ 0.8 AND ALT allele F1 ≥ 0.8), while 36.02% of SVs (SVs with REF F1 ≤ 0.2 OR ALT F1 ≤ 0.2) were incorrectly genotyped (Fig. 2C). Overall, Paragraph had a more balanced performance especially for genotyping insertions for both reference and alternative alleles (Fig. 2C). We did not observe improved performance with EVG, likely because of lack of agreement between different genotyping methods. Our results are concordant with previous findings in soybean (Lemay et al. 2022), suggesting that Paragraph is the best performing short-read graph-based genotyper also for *B. napus*. Consequently, we selected the Paragraph results for further analysis.

### Variants with good and poor genotyping outcomes have different features

**Fig. 3:**
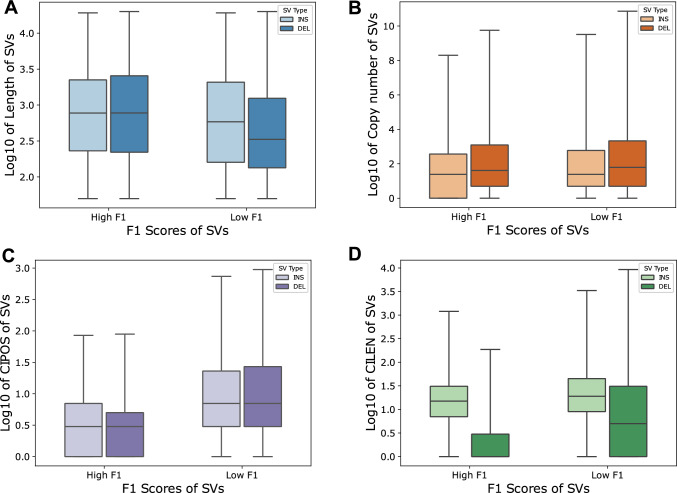
Comparison of insertion and deletion SV types with low and high F1-scores. F1-scores ≤ 0.2 are indicative of poor genotyping outcomes (Low F1), SVs with F1 ≥ 0.8 are considered correctly genotyped (High F1). **A** Comparison of length of SVs with low and high F1-score. **B** Comparison of copy number of SVs with low and high F1-score. **C** Comparison of confidence interval for position (CIPOS) of SVs with low and high F1-score. **D** Comparison of confidence interval for length (CILEN) of SVs with low and high F1-score

We further explored the reasons behind differences in F1-scores for Paragraph genotyped SVs, to understand why some variants can be genotyped with short reads while others cannot. Variants with high F1-scores for both alleles (REF allele F1 ≥ 0.8 AND ALT allele F1 ≥ 0.8) were considered correctly genotyped, while those with low F1 -scores (SVs with REF F1 ≤ 0.2 OR ALT F1 ≤ 0.2) were considered incorrectly genotyped (Fig. 2C). The correctly genotyped SVs were longer (mean: 1834.25 bp and median: 776.0 bp) compared to incorrectly genotyped SVs (mean: 1522.98 bp and median: 432.0 bp) (Fig. 3A). The correctly genotyped SVs were slightly more likely to be found in proximity of coding genes (34.1% of correctly genotyped SVs were inside or within 1 kb of protein-coding genes, compared to 33.2% for incorrectly genotyped SVs), however the overall distribution of positions relative to genes was very similar for both groups (Fig S1). The incorrectly genotyped SVs on average occurred in a higher copy number (mean: 53.81, median: 5) than the correctly genotyped SVs (mean: 44.21, median: 4) (Fig. 3B). We also found that the incorrectly genotyped SVs were associated with higher error during initial SV calling from long reads (Fig. 3C). Specifically, the incorrectly genotyped SVs had wider confidence intervals for positions (CIPOS) and lengths (CILEN) compared to correctly genotyped SVs both for deletions and insertion (Fig. 3C, D).

### SV genotyping errors are unlikely to have substantial impact on association studies

**Fig. 4:**
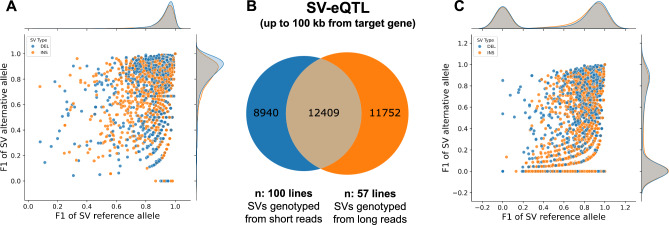
**A** Distribution of F1-scores of SVs genotyped from short reads, which were unique to SV-eQTL analysis with short-read-derived genotypes. Most of the SVs are correctly genotyped, suggesting that additional associations results from increased power (*n* = 100 for short-read genotypes vs *n* = 57 for long-read genotypes). **B** Overlap between eQTL-SVs discovered using genotyping with short (*n* = 100) and long (*n* = 57) reads. **C** Distribution of F1-scores of SVs genotyped from short reads for eQTL-SVs unique to analysis with long-read-derived genotypes. F1-scores ≤ 0.2 for either alternate or reference alleles are indicative of poor genotyping outcomes, SVs with F1 ≥ 0.8 for both alternate and reference alleles are considered correctly genotyped

To examine whether genotyping errors could affect association studies, we performed eQTL analysis using genotypes derived from 57 long-read samples and 100 short-read samples (which included the 57 samples sequenced with long reads). We compared SV-eQTL variants found within 100 kb of target genes identified in the two analyses, identifying 8,940 eQTL-SVs detected from short reads only, 11,752 eQTL-SVs from long reads only, and 12,409 overlapped eQTL-SVs (Fig. 4B). Two main sources of eQTLs unique to short-read datasets could be either the increased power of the study (57 vs 100 samples) or SV genotyping errors. Importantly, 67.02% eQTL-SVs unique to short-read analysis were determined as correctly genotyped (Fig. 4A), while only 18.67% eQTL-SVs unique to long-read analysis were determined as correctly genotyped (Fig. 4C). Overlapping eQTL-SVs from long and short reads were largely correctly genotyped: 86.69% eQTL-SVs (Fig. 4B, Fig S2). These results suggest that the eQTL-SVs identified from short reads only were mostly correctly genotyped and represented new associations found due to the increased sample size and corresponding increased power of the analysis. Conversely, eQTL-SVs found from long reads only could not be correctly genotyped from short reads and would therefore be missing from short-read only analyses. To assess the impact of the increased sample size in the short-read analysis, we downsampled the 100 short-read samples to 57 and compared variants found from the same sample size but different sequencing approaches (Fig S3). Downsampled short-read-based analysis resulted in fewer associations overall compared to the original 100 datasets and fewer associations found in SVs genotyped from short reads only.

### Genomic variation affects transcript expression quantification

**Fig. 5:**
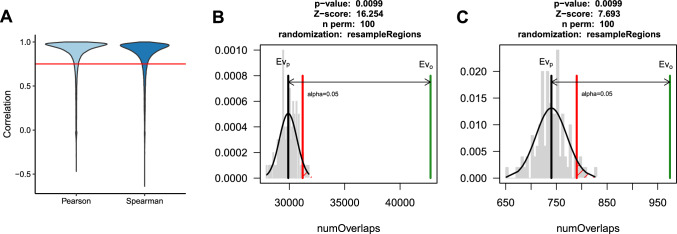
Comparison of linear reference and graph transcript expression quantification approaches. Transcripts with low concordance between Kallisto and RPVG results and overrepresented in genomic variants. **A** Pearson and Spearman correlation between Kallisto and RPVG quantification across 50 samples. Red line—0.75 cutoff used to define transcripts tested for over-representation of variants. Permutation test results: **B** Transcripts with correlation coefficient below 0.75 are significantly overrepresented in SNPs and **C** Transcripts with correlation coefficient below 0.75 are significantly overrepresented in SVs. Green line—observed value, grey line—mean of permutation results, red line—significance threshold

One of the key steps in eQTL analysis is the accurate quantification of gene expression. Sequence variation between reference genomes and the actual genotypes used for the generation of expression data can lead to quantification errors, a phenomenon often referred to as ‘reference sequence bias’ (Sibbesen et al. 2023). To test the effect of potential bias on gene expression quantification in *Brassica napus* and its impact on eQTL analysis, we compared transcript abundance derived from a linear reference based (Kallisto) and a pangenome graph-based (rpvg) approaches. The pangenome graph reference was constructed using SVs identified and genotyped from long-read data combined with SNPs called from short reads. For each transcript, we calculated the correlation between read counts estimated by the two methods across 50 samples (Fig. 5A). We then extracted transcripts with Pearson correlation below 0.75 and an equal number of transcripts with the highest correlation coefficients. If genomic variation had an appreciable effect on expression quantification, we would expect transcripts with low measurement concordance across methods to be overrepresented in variants. Indeed, we observed a statistically significant enrichment of variants in transcripts with correlation below 0.75 with a permutation test (Fig. 5B, C). Conversely, the highly correlated transcripts were depleted in variants (Figs S4A-B). A very similar result was obtained when we used transcripts per million (TPM) instead of counts as a measure of expression. To further support our observations, we simulated RNA-Seq reads for one of the samples and compared quantification results between Kallisto and rpvg quantification and expected counts for genes, which have been identified as challenging across 50 samples (correlation below 0.75). We found that the quantification results from rpvg were closer to our simulated ground truth (Fig S5). We concluded that using a pangenome graph reference could improve quantification, therefore rpvg-based expression was selected for the subsequent analysis.

### Gene-proximal structural variants are linked to gene expression regulation

**Fig. 6:**
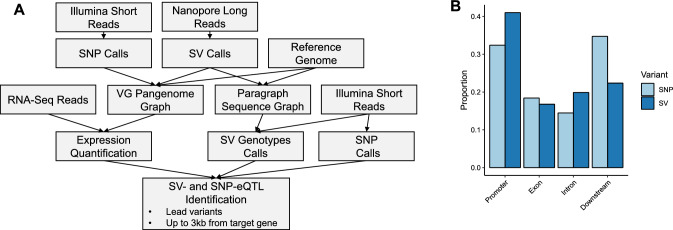
**A** Procedure for graph-based eQTL analysis: long reads are used for SV identification. SVs identified from long reads along with SNPs identified from short reads are used for graph construction. Graphs are used for transcript expression quantification and SV genotyping using a larger collection of short-read samples. **B** Distribution of eQTLs relative to genomic features highlights a higher proportion of eQTL-SVs in promoter regions compared to genic and downstream regions

Final graph-based eQTL analysis was performed using SNPs, SVs genotyped from short reads and gene expression data representing young leaves at 5–6 leaf stage from 100 homozygous inbred lines (Fig. 6A, Fig S6). In total 39,546 SVs and 2,396,948 SNPs were used in the analysis. We focused the analysis on lead eQTL variants (identified by lowest *p*-value) found in proximity of their target genes (cis-eQTLs). Due to high density of genes in the *B. napus* genome (mean distance between adjacent genes is ~ 3,500 bp) we defined cis-eQTL variants as variants located in/overlapping promoter (3,000 bp upstream from the transcription start site (TSS)), exons, introns or regions immediately downstream (3,000 bp downstream from the transcription termination site (TTS) of their target genes). Using these criteria we identified 267 SV- and 5,668 SNP-eQTLs (Supplementary Data). The proportion of SVs among eQTL variants was higher (4.7%) than among all variants used for the analysis (1.6%). For 35.1% of SV-eQTL transcripts, no significant associations between any SNP and the transcript were detected, suggesting that these eQTL-SVs are not in high enough linkage disequilibrium with SNPs to be detected in SNP-only analyses.

Within our datasets, we identified more SNP-eQTLs on the A subgenome compared to the C subgenome, while a higher proportion of SV-eQTLs was found on the C subgenome (Fig S7, Chi-squared test < 0.001).

### Majority of cis-eQTL-SVs have similarity to transposable elements

**Fig. 7:**
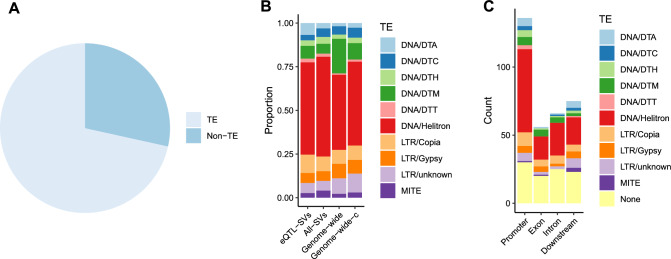
Similarity of SVs to known transposable elements. **A** Almost 70% of eQTL-SVs variants have similarity to TEs. **B** A high proportion of TE-related eQTL-SVs have similarity to Helitrons compared to Genome-wide (based on counts of TEs annotated by GenomeMasker) and Genome-wide-c (based on counts of TEs annotated by GenomeMasker after merging overlapping elements of the same family). **C** A high number of promoter-associated eQTL-SVs has similarity to Helitrons compared to eQTL-SVs related to other genomic features

We investigated the distributions of eQTL-SNPs and SVs in relation to transcript feature locations. Compared to SNPs, a higher proportion of eQTL-SV were found in promoters (Fig. 6B, Chi-Square *p* < 0.01). Overall, a high relative prevalence of SVs upstream of the TSS was previously observed and linked to Class II (DNA) transposable element activity, which can perhaps be explained by easier accessibility of these regions (Han et al. 2013; Fuentes et al. 2019). Indeed, we observed that 71% of eQTL-SVs have similarity to DNA transposable elements (Fig. 7A).

**Fig. 8:**
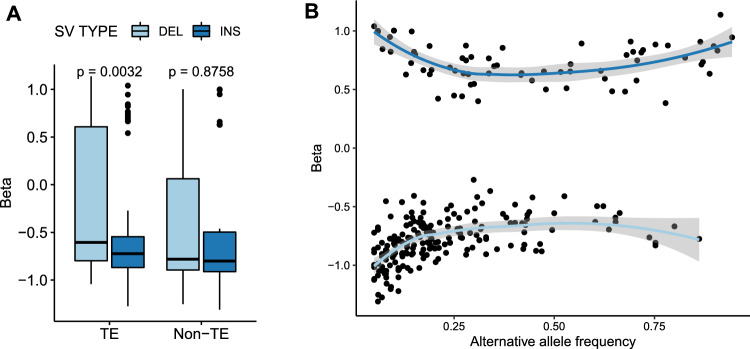
Effect size of eQTL-SVs. **A** TE insertions are associated with decreased expression compared to deletions. **B** SVs with higher effect size have a lower frequency in the population

We found that 56% of eQTL-SVs have similarity to Class II (DNA) transposons, 15% to Class I (RNA) transposons and 29% had no detectable similarity to TEs identified in the *B. napus* genome. Among the TE-related eQTL-SVs, the most common TE family was Helitron. The proportion of Helitrons among eQTL-SVs was higher than observed genome wide, but similar to all SVs (Fig. 7B, Fig S8). Helitrons were also the most abundant class of TEs found in promoter-located eQTL-SVs (Fig. 7C). Overall, TE insertions had a greater negative impact on gene expression than deletions (Fig. 8A, Fig S9). Together these results suggest that transposable elements, especially Helitrons, contribute to gene expression diversity in *B. napus*.

Previous eQTL studies reported a relationship between effect size (Beta) and allele frequency, with SVs associated with higher effect sizes found at lower frequencies in the population (Uzunović et al. 2019; Castanera et al. 2023). We observed a similar pattern in our data for both SVs (Fig. 8B, Fig S10A-D) and SNPs (Fig S11). These results are in line with the expected deleterious effects of rare alleles (Lye et al. 2022) and further support the high quality of our variant and eQTL calls. We observed no significant difference in Beta (Fig S12) and variance (Fig S13) explained by lead eQTL-SNPs and SVs.

### Selected examples of genes affected by eQTL-SVs

**Fig. 9:**
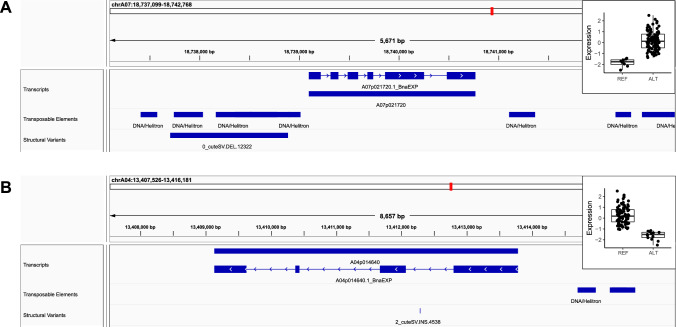
Example of SVs associated with different gene expression levels. **A** Deletion of Helitron TE in the promoter region of A07p021720 is associated with an increase in gene expression. The corresponding Arabidopsis homologue (SNRK2.8) is known to be involved in response to osmotic stress (TAIR). **B** Insertion of Helitron TE in the first intron of A04p014640 is associated with decreased expression. Arabidopsis homologues (SAW2) is involved in leaf morphogenesis (TAIR). Expression is reported after inverse normal transformation. REF = reference allele, ALT = alternate allele

Out of 259 SV-eQTL transcripts identified, 92% had homologues in the Arabidopsis genome. Gene ontology enrichment analysis did not indicate over-representation in specific processes or functions. However, some transcripts were annotated with functions related to important traits, including stress response (Fig. 9A) and morphogenesis (Fig. 9B). These results suggest that SV-driven gene expression variation could contribute to the phenotypic diversity observed in the field.

## Discussion

We used a pangenome graph approach to discover SNPs and SVs associated with differences in gene expression in young leaves of winter oilseed rape. The pangenome graph was used for structural variant genotyping, but also gene expression quantification. We showed that SVs discovered from long reads mostly either genotype correctly from short reads or fail to genotype altogether, and genotyping errors are therefore unlikely to lead to false associations. However, a failure to genotype does reduce the pool of SVs available for association studies, as exemplified here, with approximately half of the initially discovered SVs being successfully genotyped from short reads and included in the downstream eQTL analysis. Failure to genotype was associated with features such as SV length (shorter SV were more difficult to genotype) and higher uncertainty of the initial SV call. It is important to note that, due to its allotetraploid genome, *B. napus* represents a particularly challenging case of SV genotyping and graph construction, as tools for these analyses were predominantly tested on diploids. Our results are in line with previous reports that SV genotyping had lower performance in paleopolyploid soybean compared to diploids, likely due to ambigious mappings of short reads across sub-genomes (Chen et al. 2019; Sirén et al. 2021; Lemay et al. 2022; Du et al. 2024).

We used medium sequencing coverages (> 10x) for both short and long reads. While this coverage is insufficient for the recent pangenome graphs building pipeline (PGGB, Garrison et al. 2018, Kopalli et al., in preparation), which requires multiple whole genome assemblies, it is appropriate for alignment-based SV discovery combined with VG pangenome graphs. We achieved reasonably good precision and recall at SV calling despite the limited coverages, as anticipated based on our previous study (Yildiz et al. 2023), which demonstrated the identification of SVs using medium-depth (5x-20x) Oxford Nanopore reads. The main limitation of using mid-coverage ONT data is a limited accuracy in the identification of exact break points and deriving consensus sequences, which appears to be reflected in genotyping results. Even using a graph-based approach, SVs with less confident breakpoints and insertions were more difficult to genotype. With the latest improvement in sequencing technologies, resulting in reduced error rates for both ONT and PacBio long reads, high quality calls will be achieved even at low to moderate coverages.

We found that the majority of identified *B. napus* eQTL-SVs sequences have similarity to transposable elements. The finding is in line with reports in other crops, including rice and *B. rapa,* where transposable element insertion polymorphisms (TIPs) were shown to contribute to phenotypic and gene expression variation (Cai et al. 2022; Castanera et al. 2023). Approximately 29% of eQTL-SVs were not annotated as TEs in this analysis. While this could be partly due to current limitations of TE detection tools (Loreto et al. 2023), we observed a more negative effect of insertions annotated as TEs compared to non-TE ones (Fig. 8), suggesting that the latter are truly not TE derived.

Compared to eQTL-SNPs, eQTL-SVs are more likely to be found in promoter regions of genes. The preference of certain transposable elements for insertion into open chromatin regions and especially promoters could make them particularity suited for the rewiring of regulatory networks (Fuentes et al. 2019; Cao et al. 2023; Barro-Trastoy and Köhler 2024). In *B. napus,* the highest number of eQTL-SVs had sequence similarity to Helitrons, likely reflecting their overall high abundance in the genome, where they cover approximately 20% of the genome and represent approximately 50% of all annotated TEs. However, many of SVs with similarity to Helitrons appear to represent TE fragments rather than intact elements. Previous studies confirmed that Brassicas carry a high abundance of Helitrons relative to other tested species (Hu et al. 2019). While retro-transposons are more abundant in centromeric regions, distribution of DNA elements including Helitrons mirrors more closely the distribution of genes (Fig S8), reflecting their potential for altering gene activity. For example, Helitrons have been shown to play important roles in modifying gene regulation in genes involved in endosperm development and response to herbivory (Barro-Trastoy and Köhler 2024). In addition, the higher prevalence of eQTL-SVs upstream of the TSS can perhaps be explained by easier accessibility of these regions resulting in preferential TE insertion (Han et al. 2013; Fuentes et al. 2019).

Overall, TE insertions had a more negative impact on gene expression than TE deletions. This pattern was not observed for non-TE SVs. The presence of TEs is known to be associated with transcription factors activity disruption and increased DNA methylation, which can have a silencing effect on gene expression (Hollister and Gaut 2009). The stronger negative effect of TE insertions suggests that, at least to some extent, epigenetic silencing mechanisms may be at play.

Functional annotation of SV-eQTL transcripts suggests the involvement of some SVs in modulating important biological processes such as stress responses, flowering and morphogenesis. Due to the highly duplicated nature of the *B. napus* genome, owing to whole genome triplication in the ancestral species of *Brassica* and a more recent allopolyploidization (Cheng et al. 2014), predicting the impact of SVs on traits is not straightforward, even when associated with gene expression differences. Encouraging examples nevertheless exist. For example, despite the presence of multiple homologues, a deletion within the second intron of a *B. napus* FLOWERING LOCUS T homologue was associated with altered flowering time (Vollrath et al. 2021).

Our study highlights the contribution of structural variations to gene expression regulation and the utility of pangenome graph for eQTL analyses in crops. Even using a moderate sample size (*n* = 100) we identified an appreciable number of SVs associated with differences in gene expression. Expanding the sample size and including additional organs and developmental stages will likely result in the identification of many more SVs affecting gene regulation and, potentially, favourable agronomical traits.

## Conclusion

In this study, we combined long- and short-read whole genome sequencing data with expression profiling of *Brassica napus* leaves to assess the impact of structural variants (SVs) on gene expression regulation and explore the utility of pangenome graphs for expression quantitative trait locus (eQTL) mapping. Using the graphical pangenome reference for both expression quantification and SV genotyping, we found that insertions, deletions and especially transposable elements (TEs) contribute to gene expression diversity in *B. napus* and that a high proportion of potentially functionally important SVs are not in linkage disequilibrium with SNPs. These SVs affect expression of genes related to important traits and represent diversity unaccounted for in classical SNP-based analyses, highlighting the still largely untapped potential of SVs in eQTL studies.

## Supplementary Information

Below is the link to the electronic supplementary material.

Supplementary file1 (DOCX 6860 KB)

## Data Availability

All raw data generated in this study have been submitted to the NCBI BioProject database (https://www.ncbi.nlm.nih.gov/bioproject/) under accession number PRJNA1086556. It is made available under a CC-BY-NC-ND 4.0 International licence. Supplementary Data: eQTL results and corresponding variants can be accessed under: https://osf.io/gfphb/
