# Supplementary material for: Graphical pangenomics-enabled characterization of structural variant impact on gene expression in Brassica napus
Source: Theor Appl Genet. 2025 Apr 3;138(4):91. doi: 10.1007/s00122-025-04867-2 (PMC11968540; doi:10.1007/s00122-025-04867-2)
Supplement: Supplementary file 1 — Supplementary file1 (DOCX 6860 KB) [file 122_2025_4867_MOESM1_ESM.docx]

**Supplementary material**

**
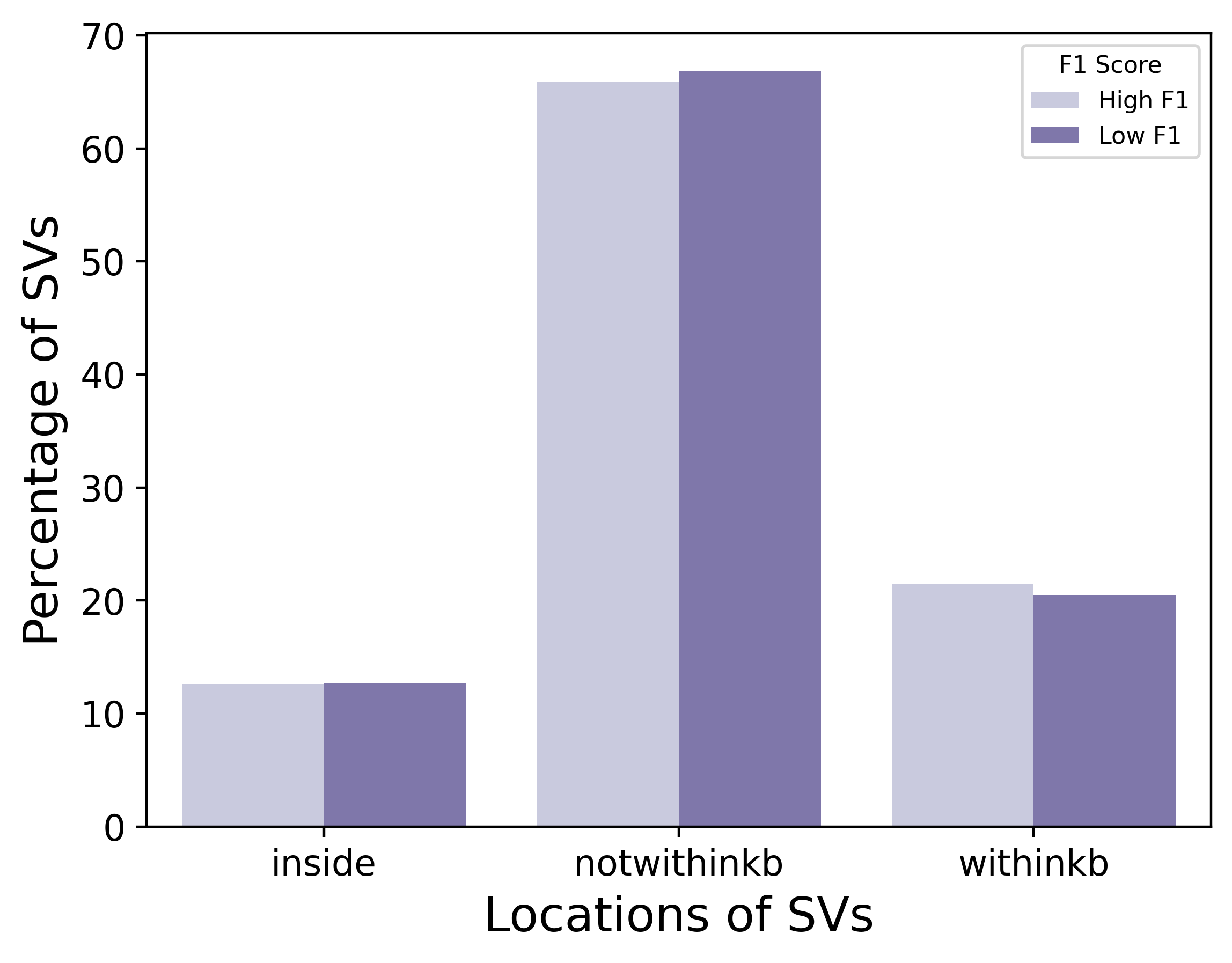
**

**Figure S1.** Gene-Proximity patterns for variants with high and low F1 scores with Paragraph.

**
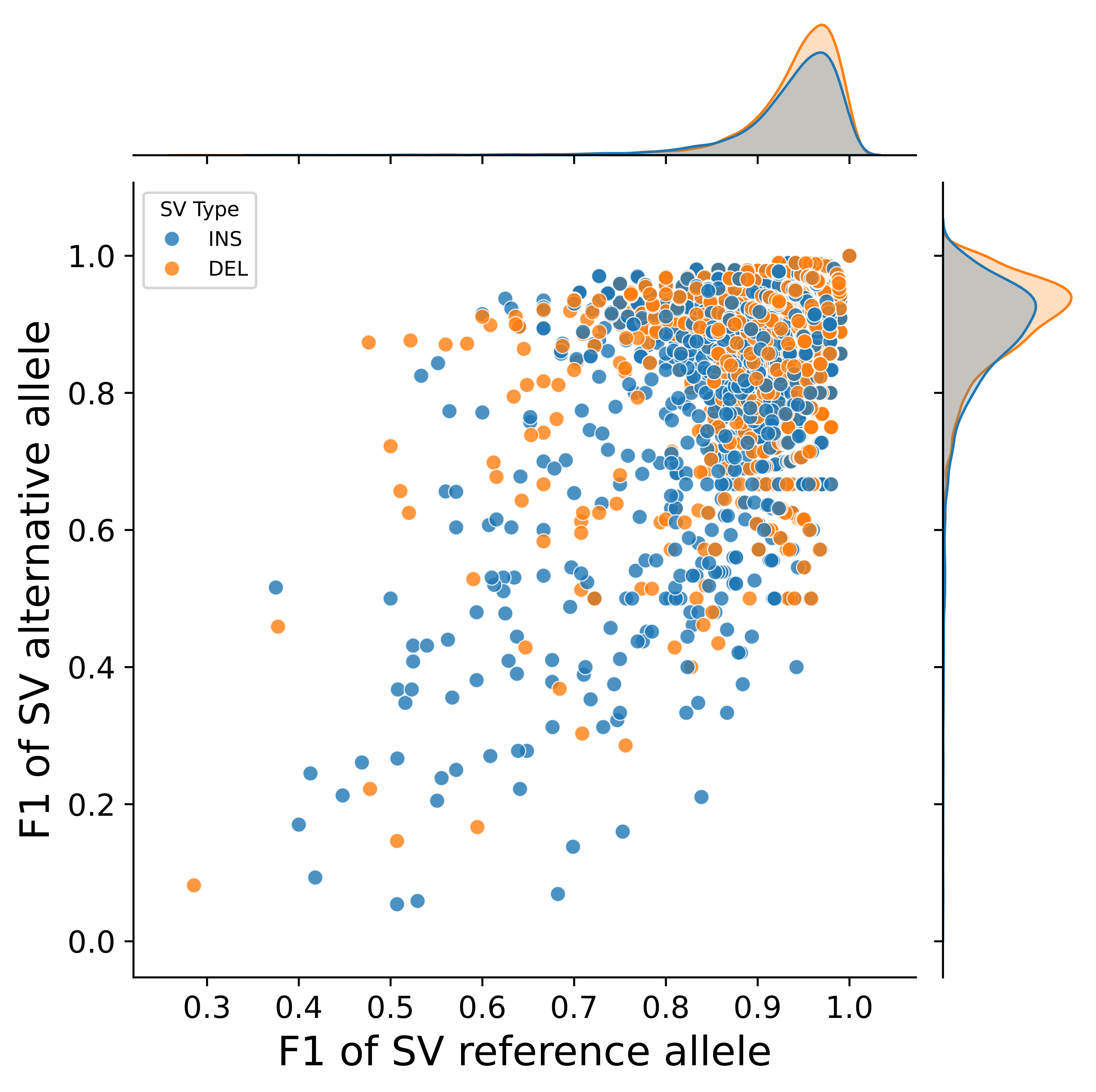
**

**Figure S2.** Distribution of F1-scores of overlapped SVs between eQTL-SVs discovered using genotyping with short (n=100) and long (n=57) reads.

**
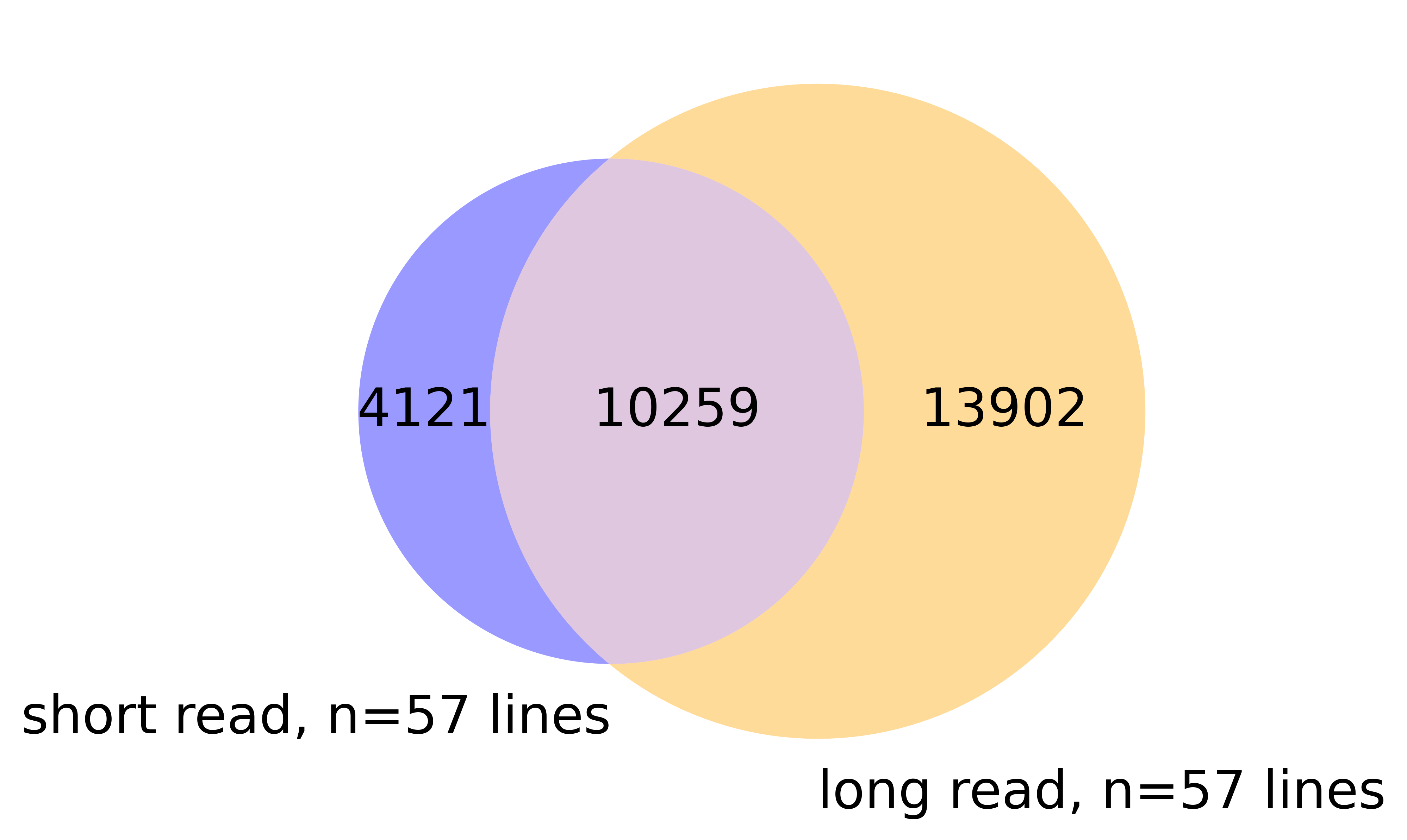
**

**Figure S3A: Venn diagram illustrating the overlap and unique eQTL-SVs identified through genotyping using short-read (n=57) and long-read (n=57) sequencing.**

**
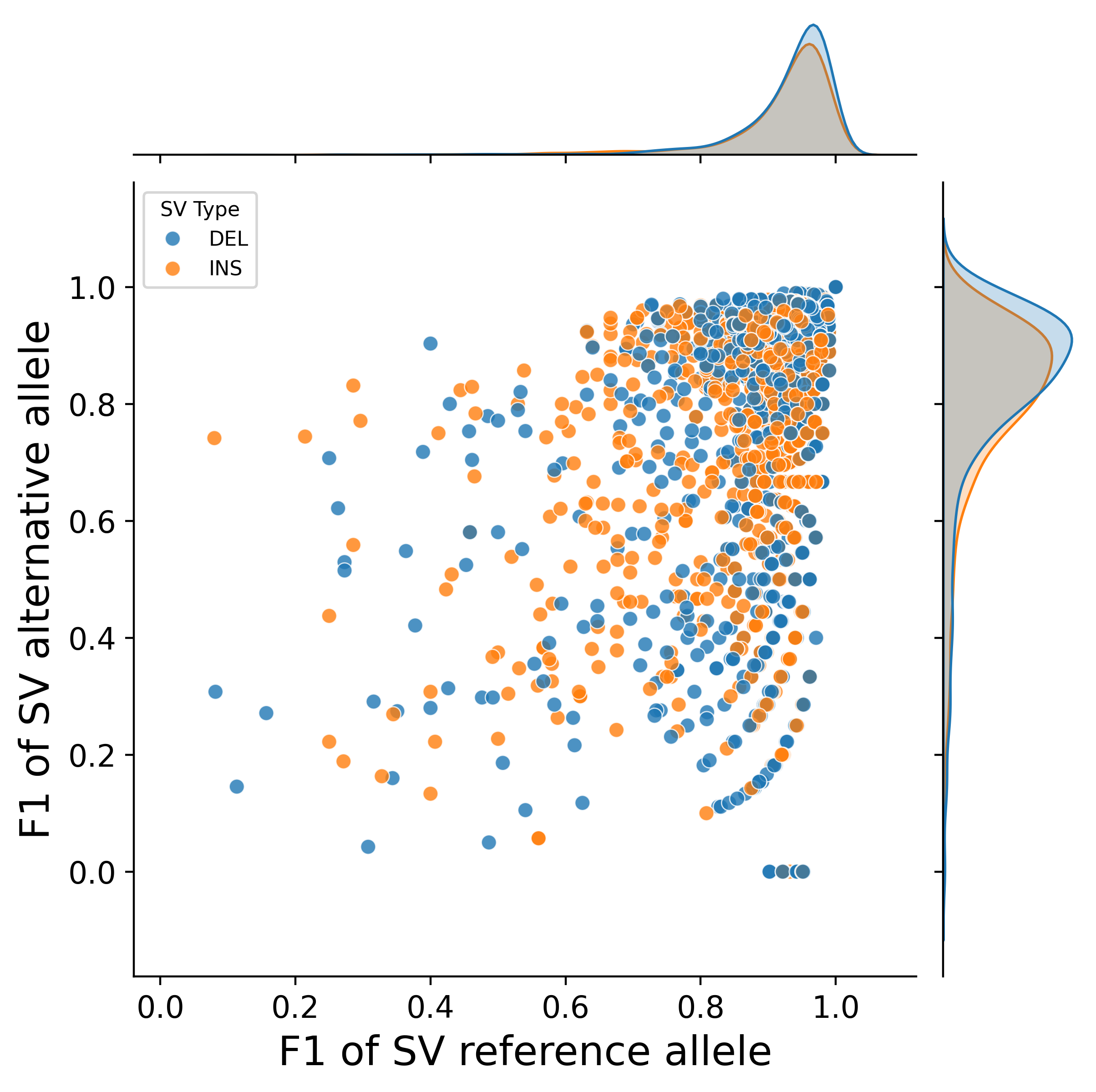
**

**Figure S3B: Distribution of F1-scores of SVs genotyped from short reads, which were unique to SV-eQTL analysis with short read-derived genotypes (n:57 short reads).**

**
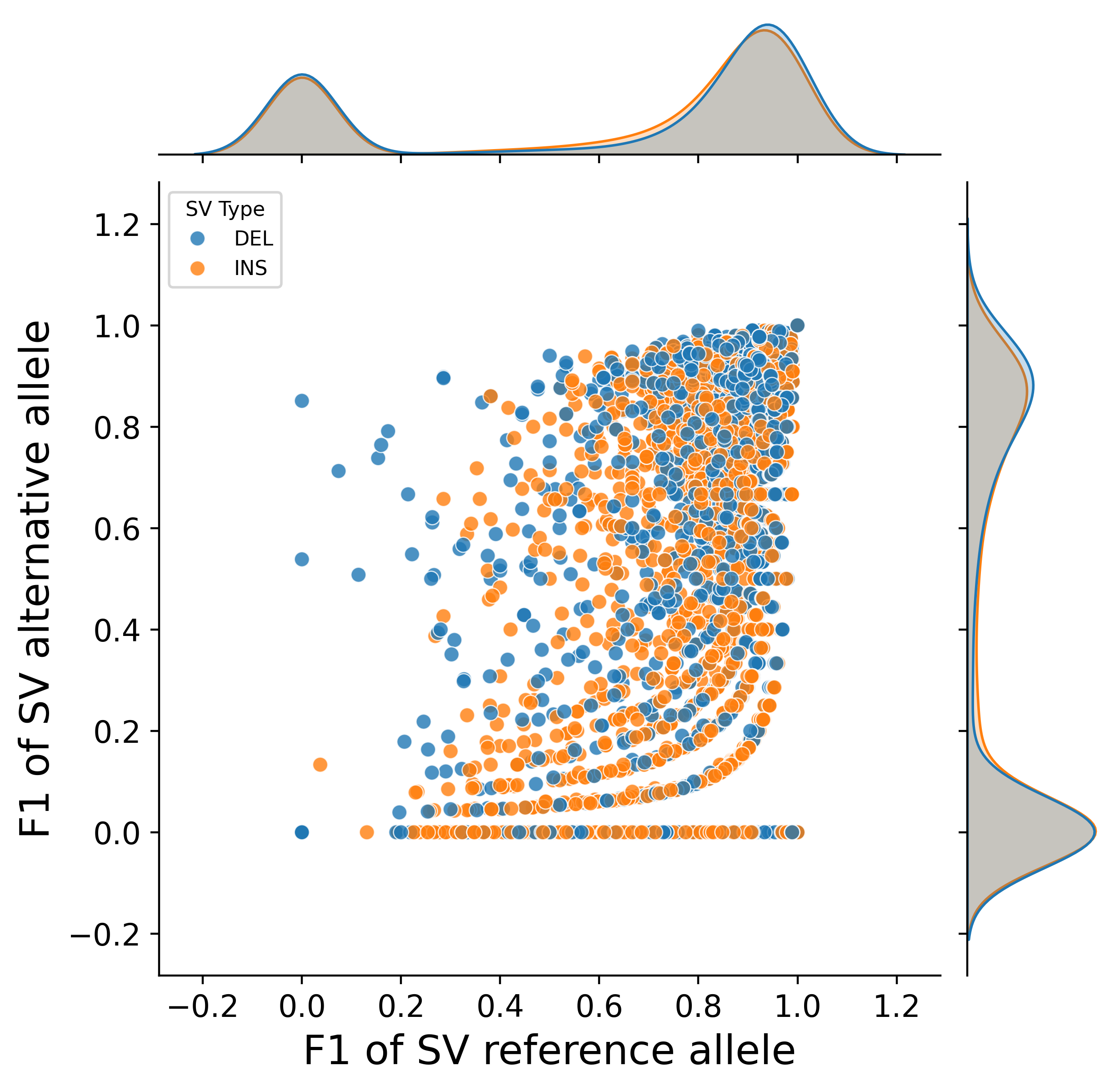
**

**Figure S3C: Distribution of F1-scores of SVs genotyped from short reads for eQTL-SVs unique to analysis with long read-derived genotypes (n:57 long reads).**

**
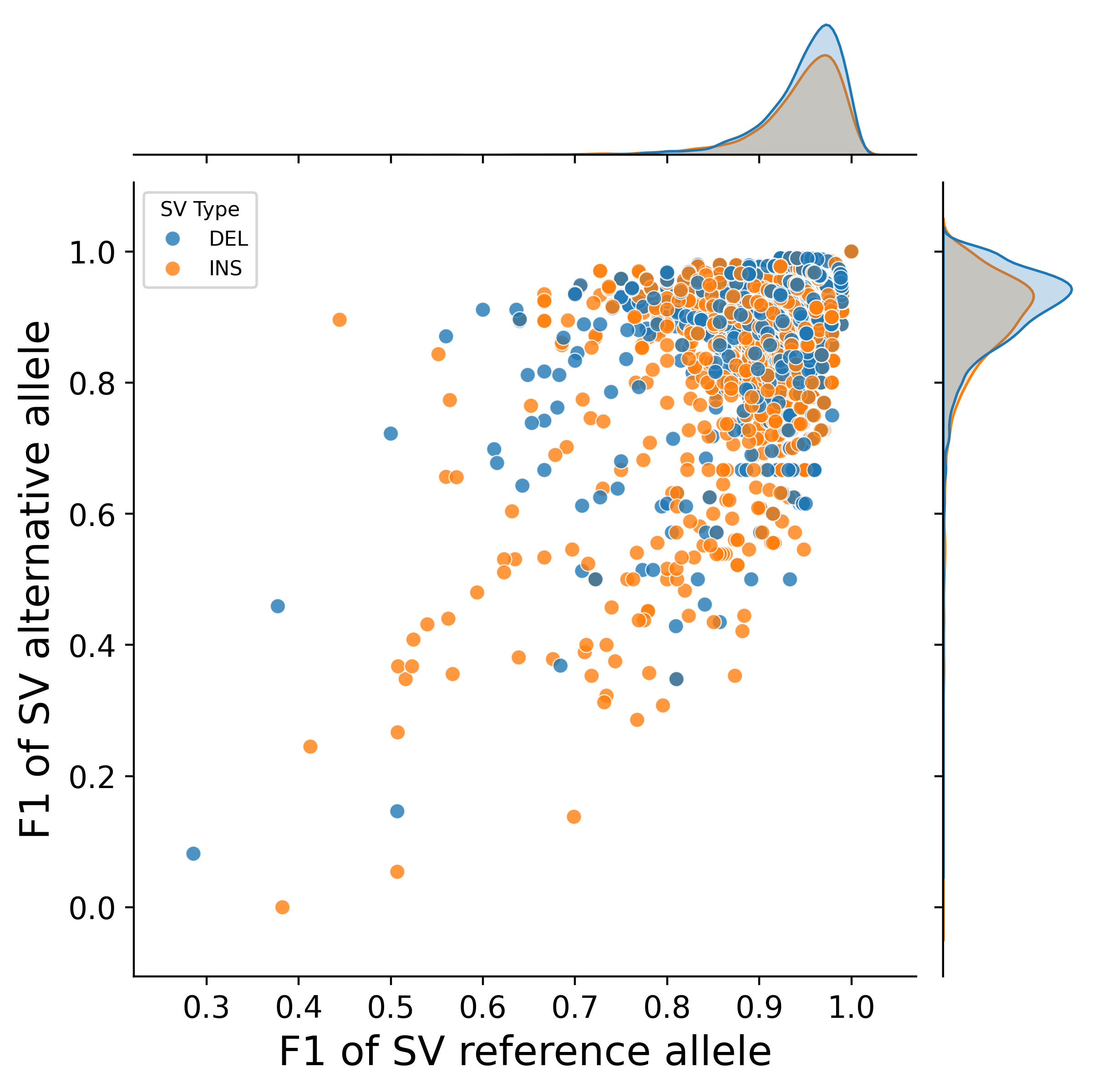
**

**Figure S3D: Distribution of F1-scores of overlapped SVs between eQTL-SVs was discovered using genotyping with short (n:57) and long (n:57) reads.**


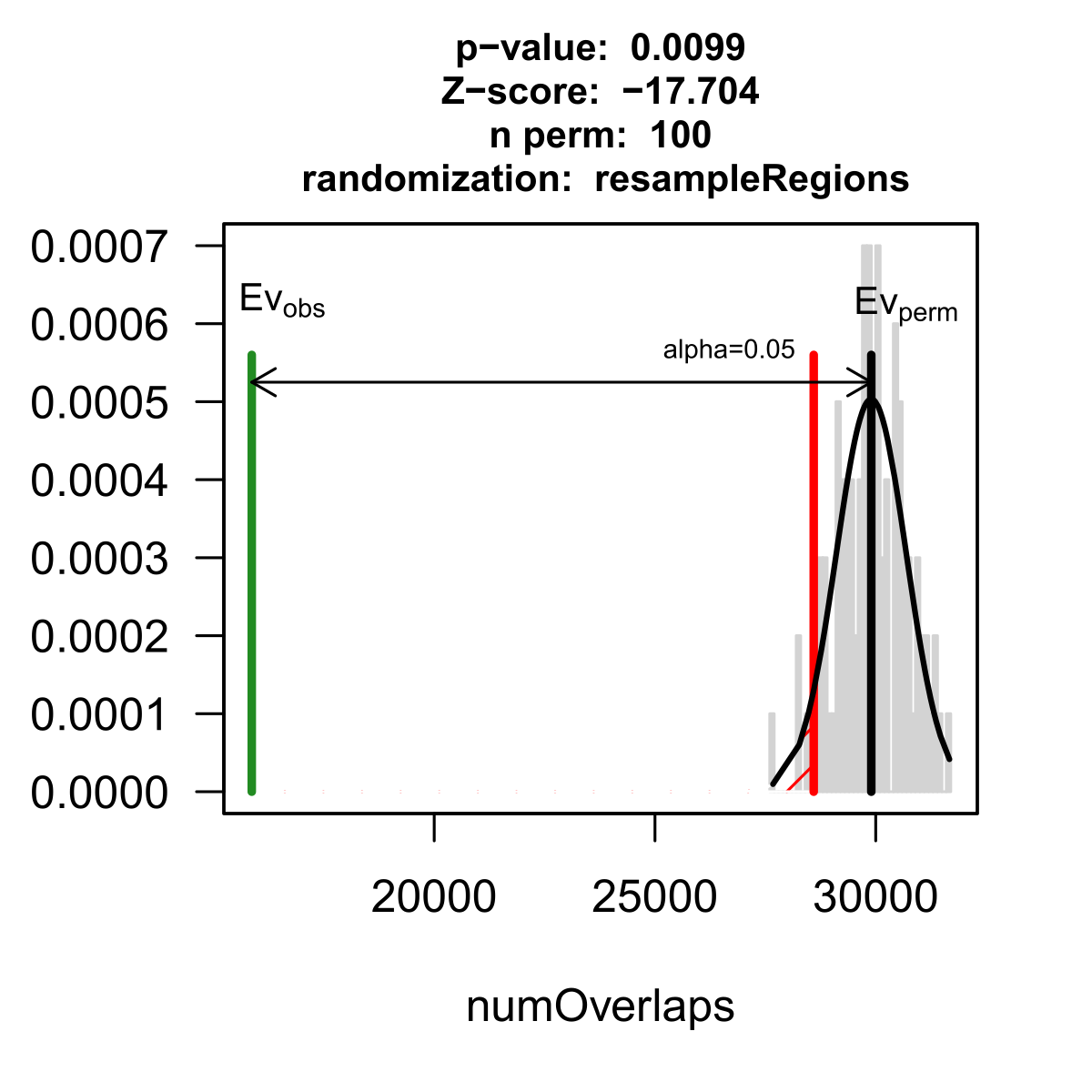


**Figure S4A**. Transcripts with high concordance of quantification results from Kallisto and RPVG are under-represented in SNPs. Green line – observed value, grey line - mean of permutation results, red line – significance threshold.


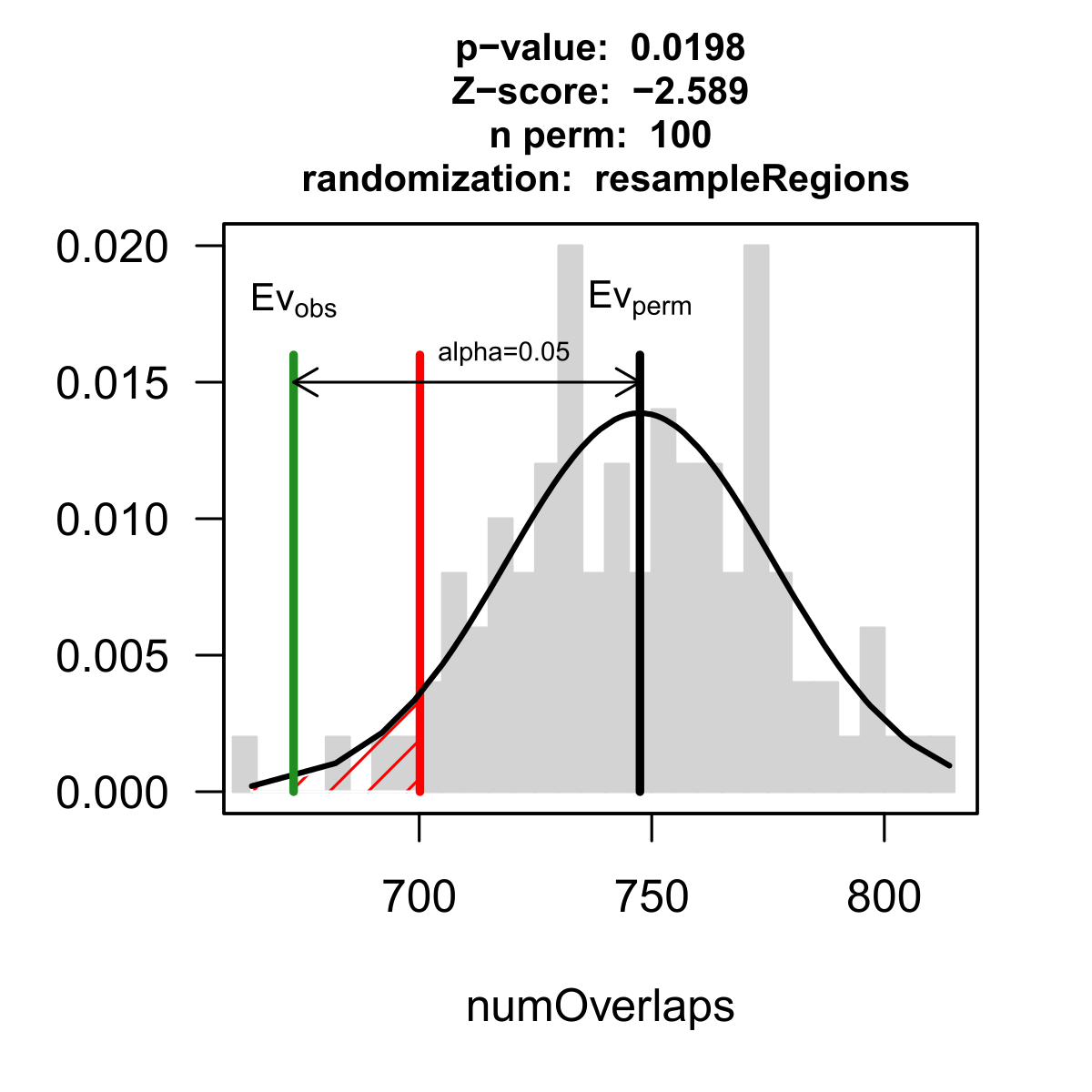


**Figure S4B**. Transcripts with high concordance of quantification results from Kallisto and RPVG are under-represented in SVs. Green line – observed value, grey line - mean of permutation results, red line – significance threshold.


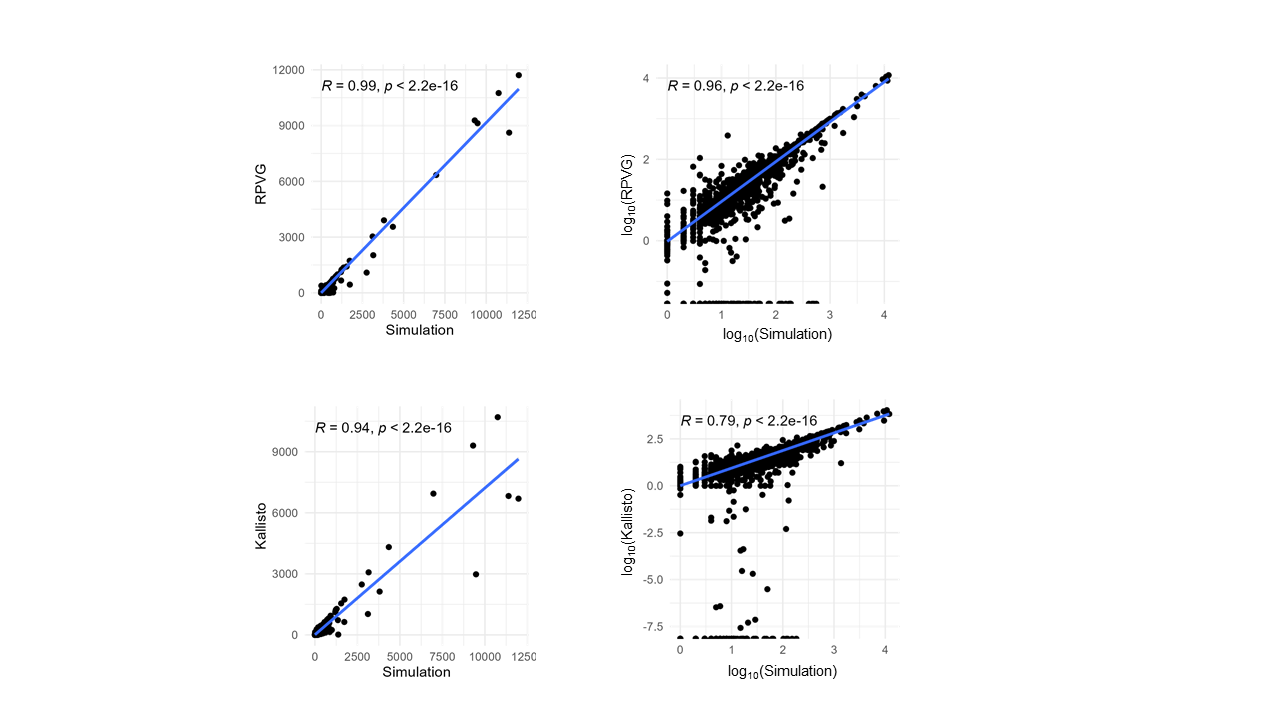


**Figure S5**. Correlation between simulated read counts and quantification results from rpvg and Kallisto.

**
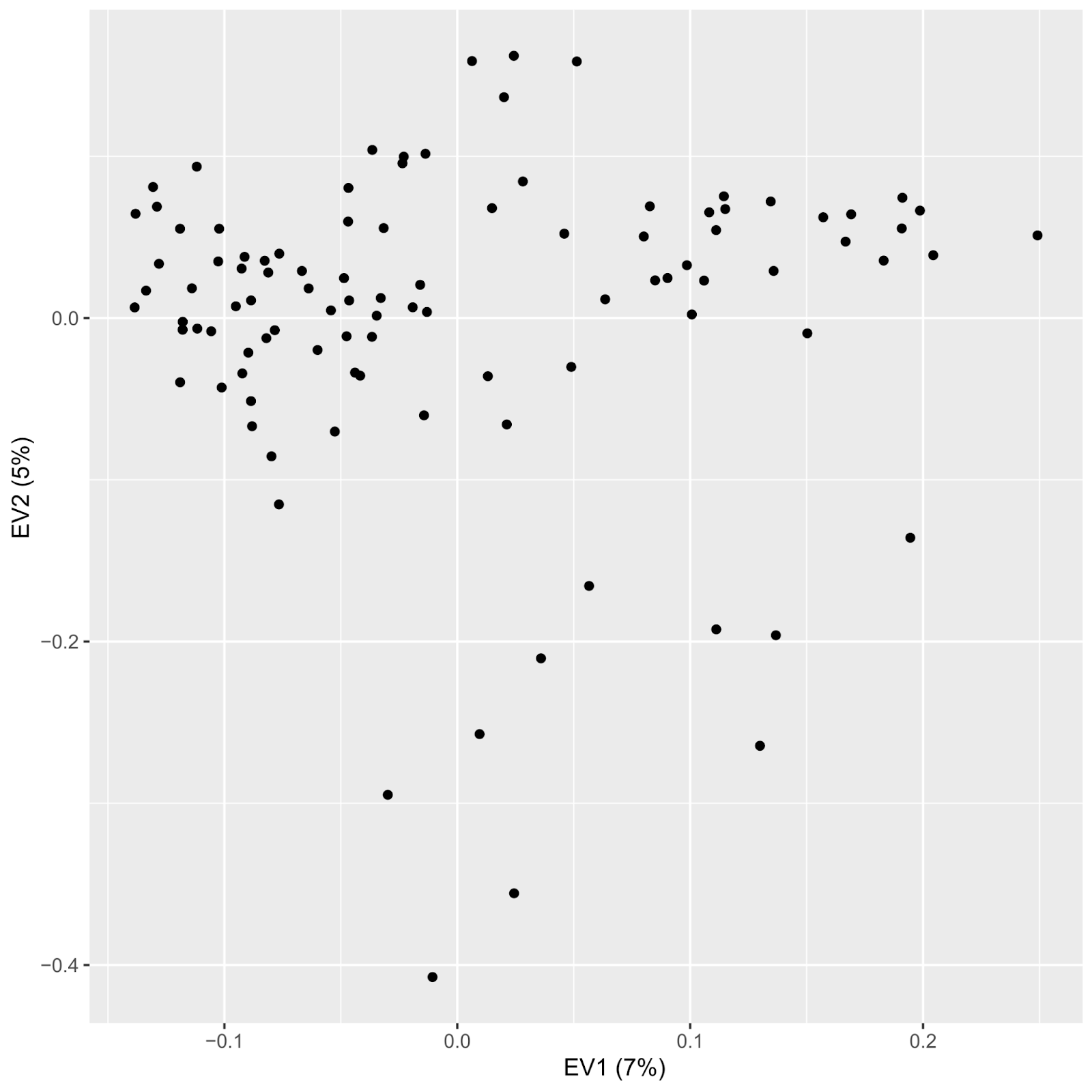
**

**Figure S6A: Principal Component Analysis (PCA) plot of SNPs (n: 100) discovered based on linear reference.**

**
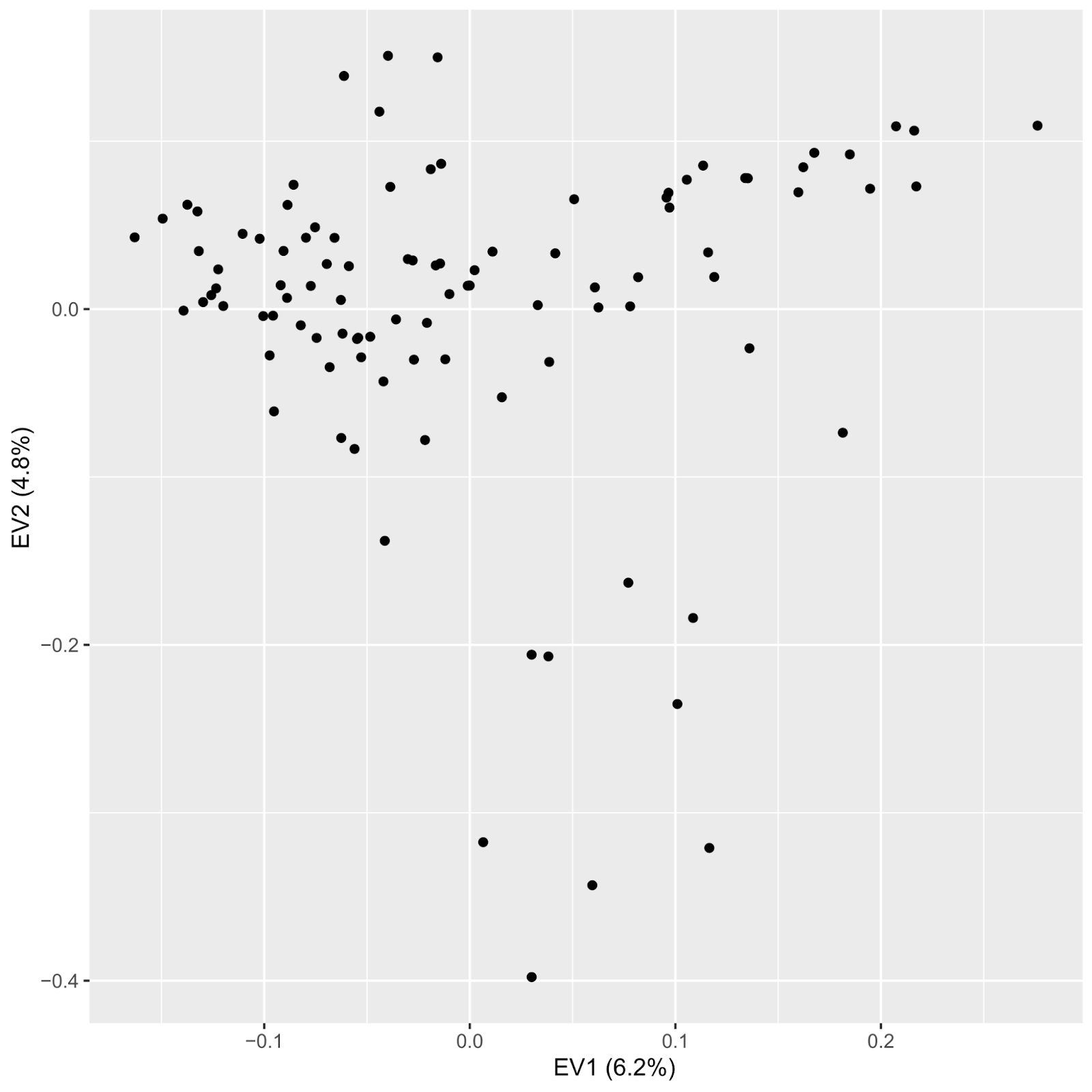
**

**Figure S6B: Principal Component Analysis (PCA) plot of SVs (n: 57) genotyped from graph.**

**
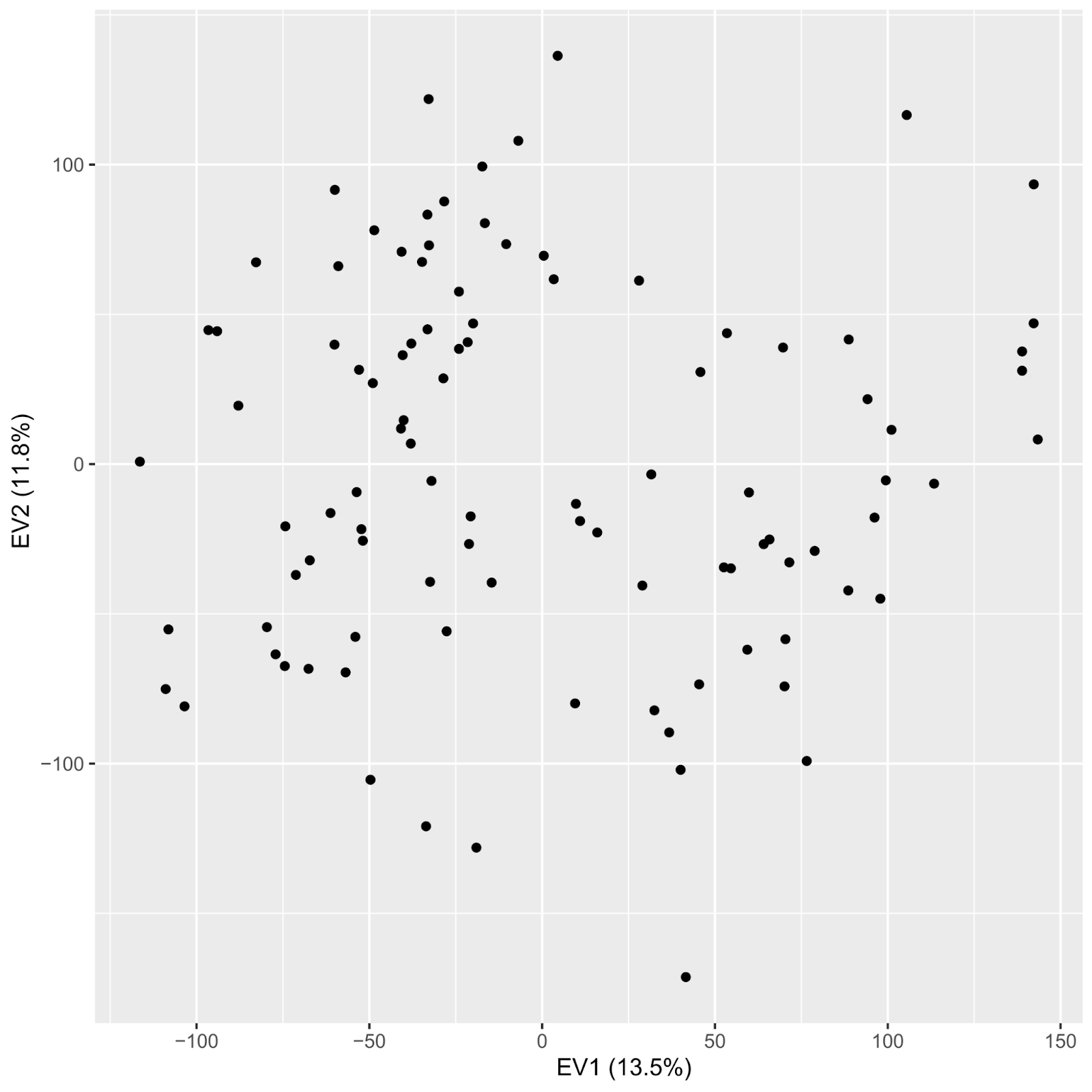
**

**Figure S6C: Principal Component Analysis (PCA) plot of RNASeq (n: 100) quantified from graph.**


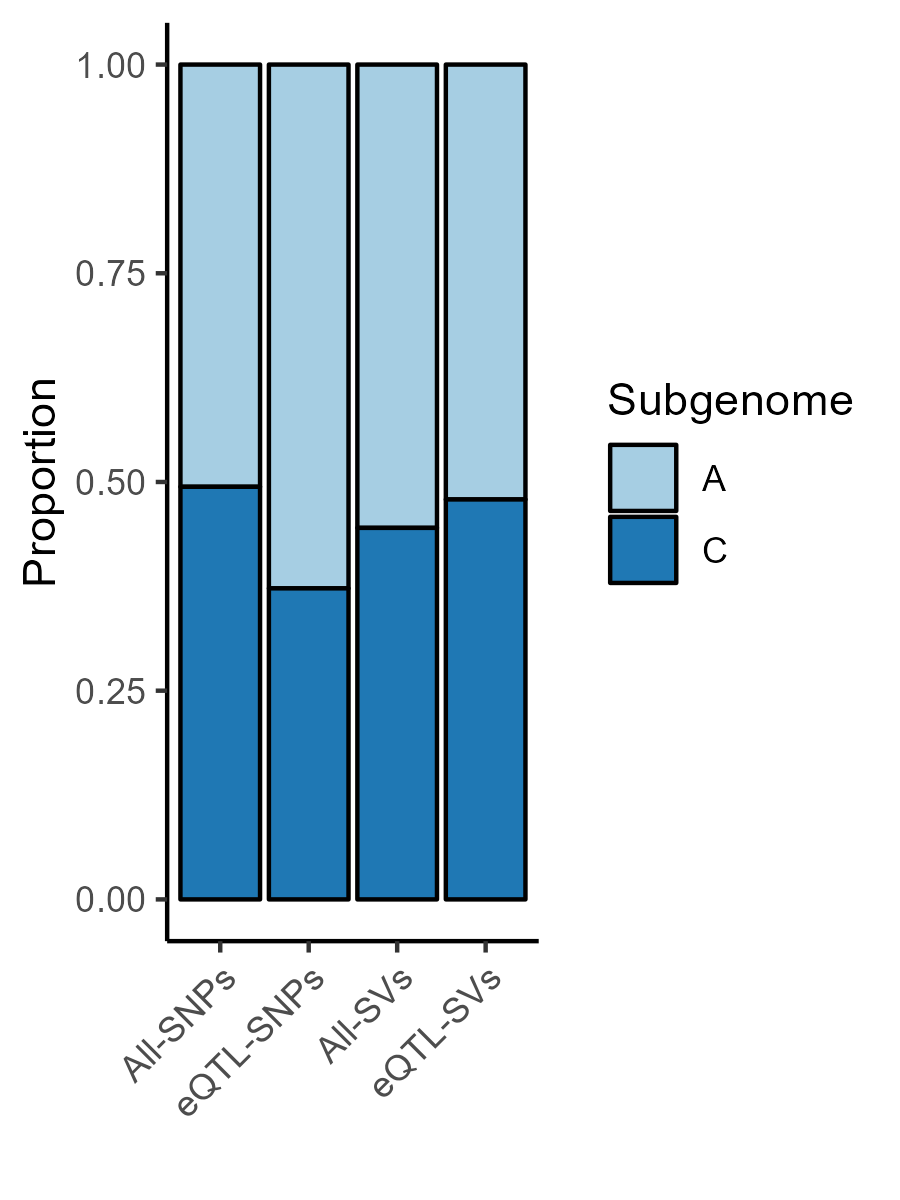


**Figure S7:** Distribution of eQTLs relative to genomic features. eQTL-SNPs and eQTL-SVs are differently distributed across the two sub-genomes of *Brassica napus*.

**
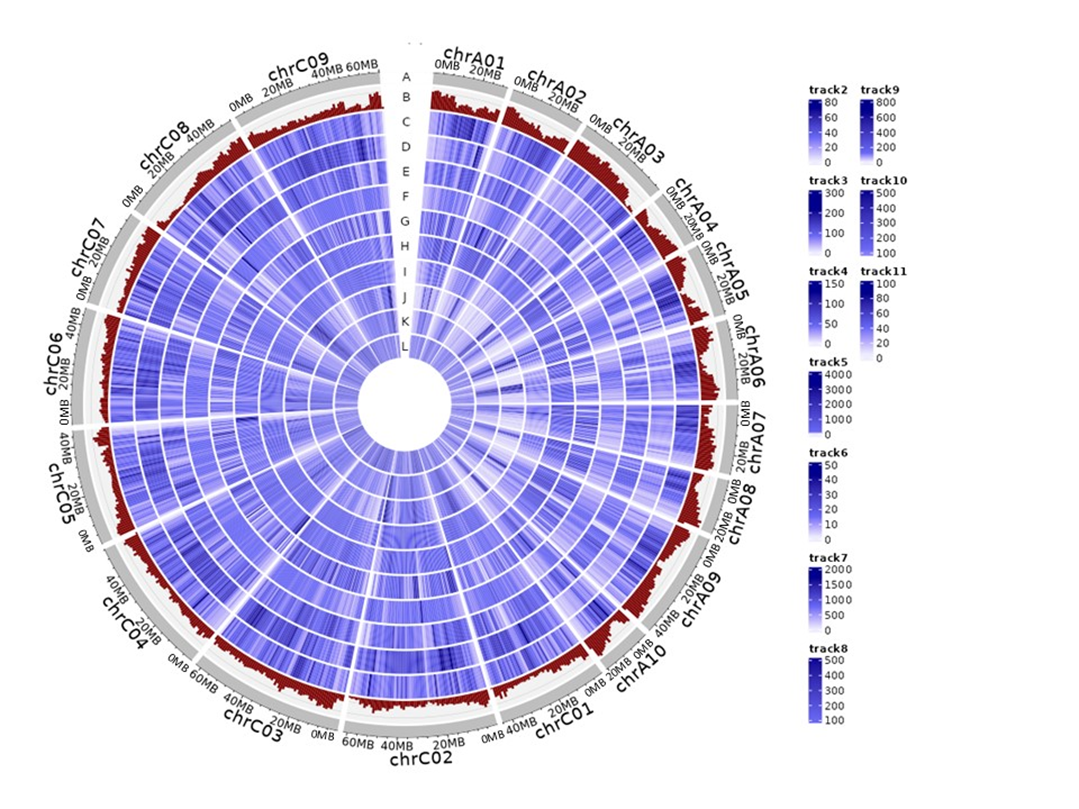
**

**Figure S8.** Distribution of transposable elements (TEs) abundance of the *B. napus* genome. Chromosomes (A), Gene density (B), DNA/DTA TEs density (track 2) (C), DNA/DTC TEs density (track 3) (D), DNA/DTH TEs density (track 4) (E), DNA/DTM TEs density (track 5) (F), DNA/DTT TEs density (track 6) (G), DNA/Helitron TEs density (track 7) (H), DNA/Copia TEs density (track 8) (I), LTR/Gypsy TEs density (track 9) (J), LTR/unknown TEs density (track 10) (K), MITE TEs density (track 11) (L). Densities were calculated using 1 Mb window size.


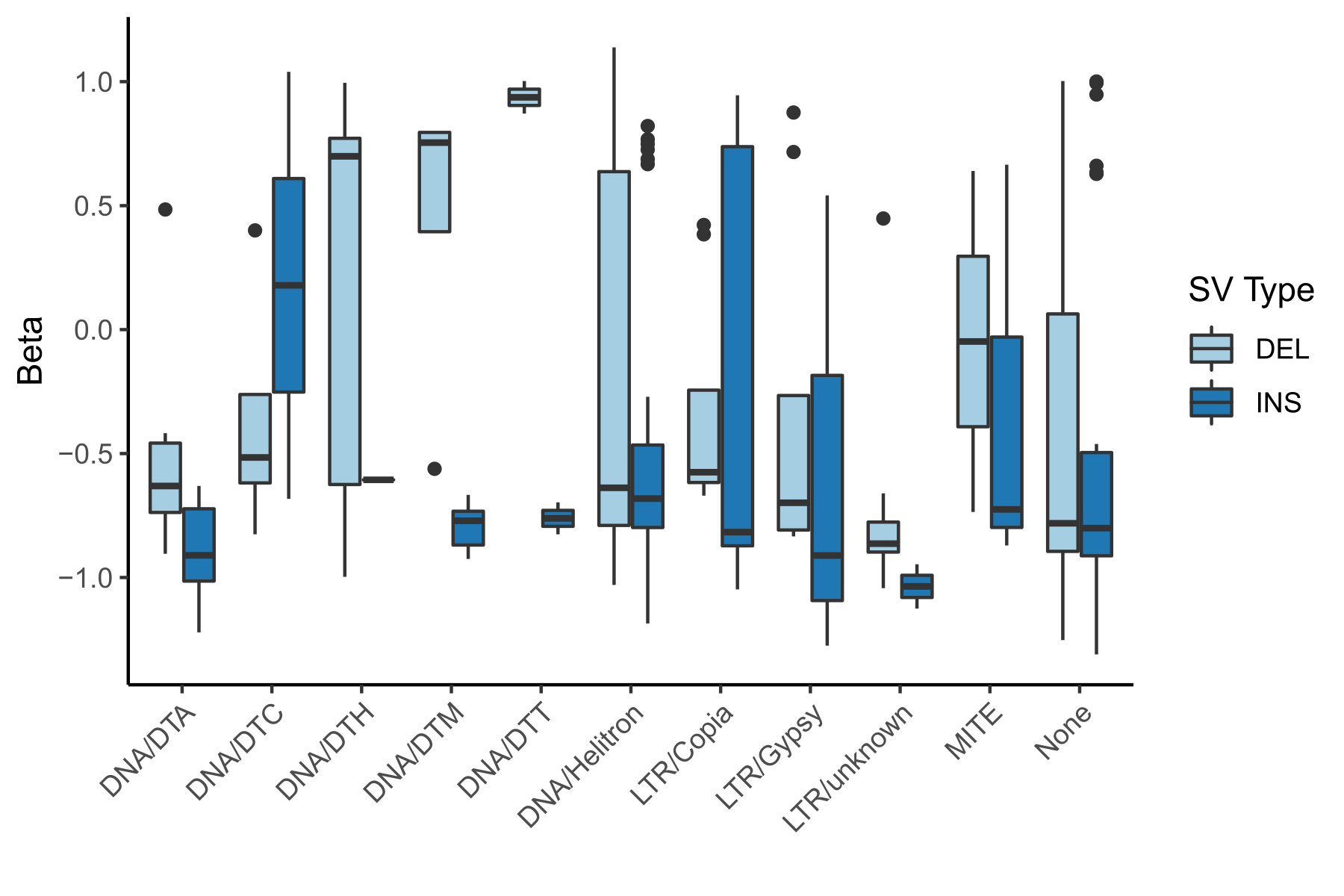


**Figure S9**. Effect of sequence insertions and deletions on gene expression.


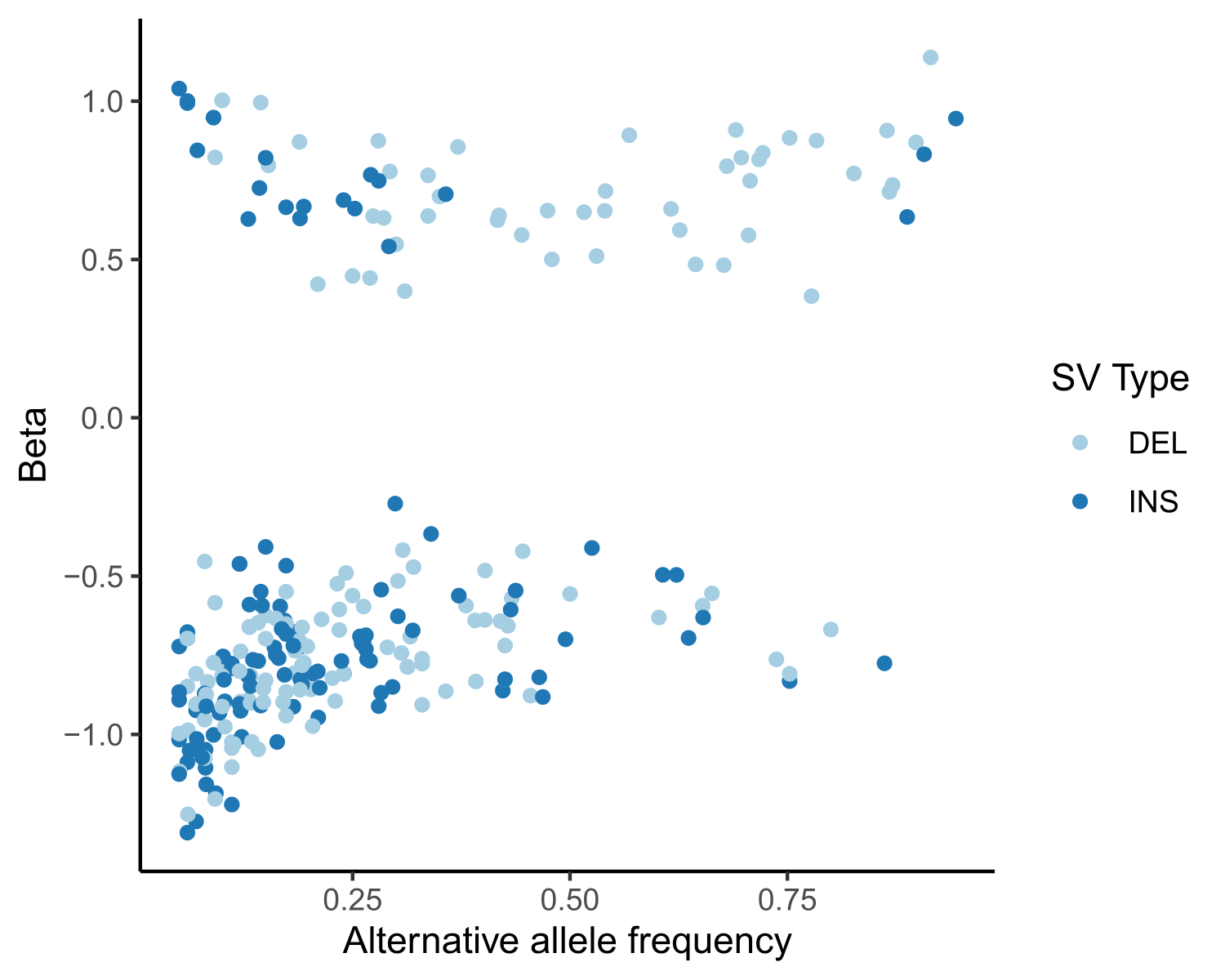


**Figure S10A**. Relationship between SV alternative allele frequency, effect size (Beta) and variant type.


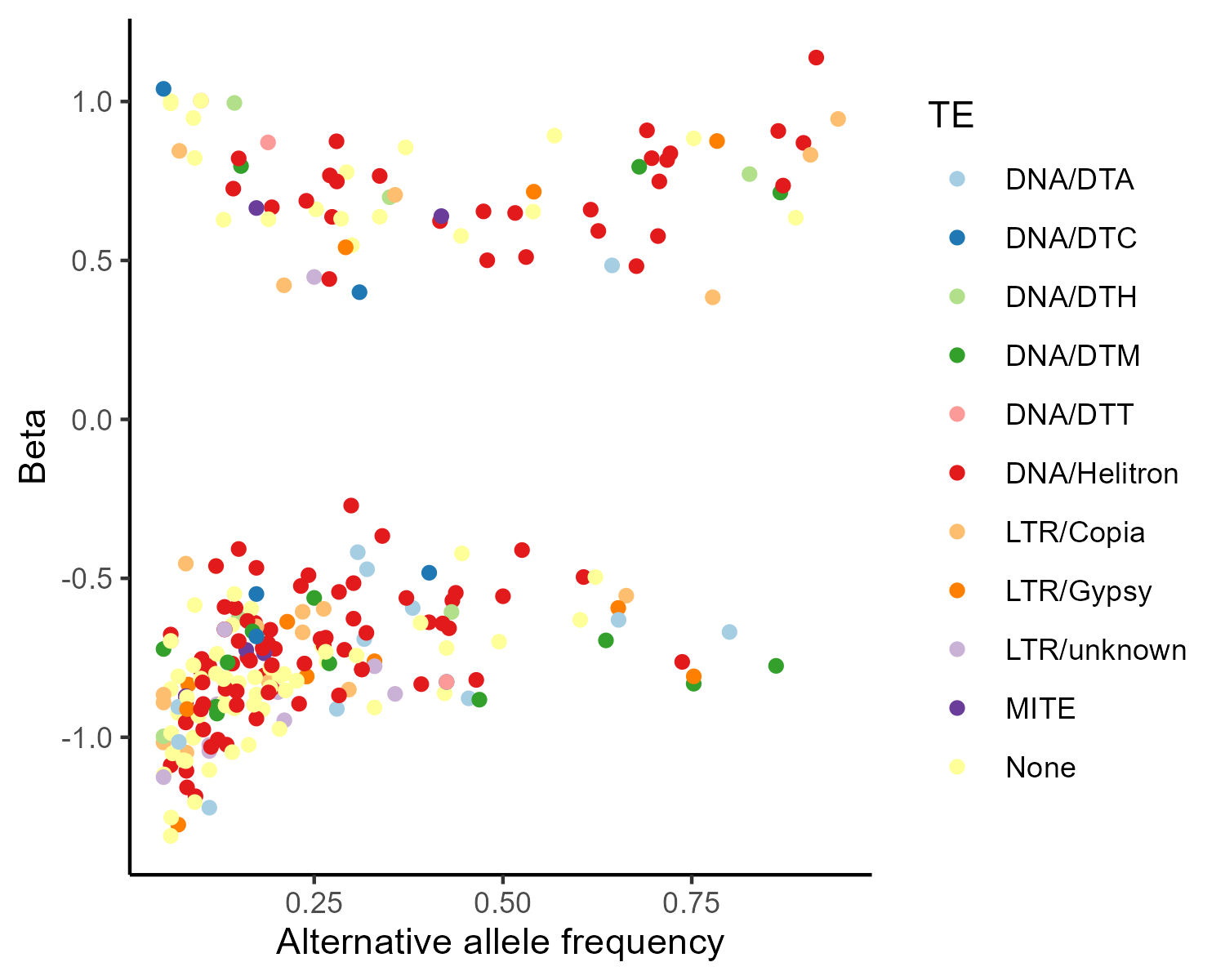


**Figure S10B**. Relationship between SV alternative allele frequency, effect size (Beta) and TE classification.


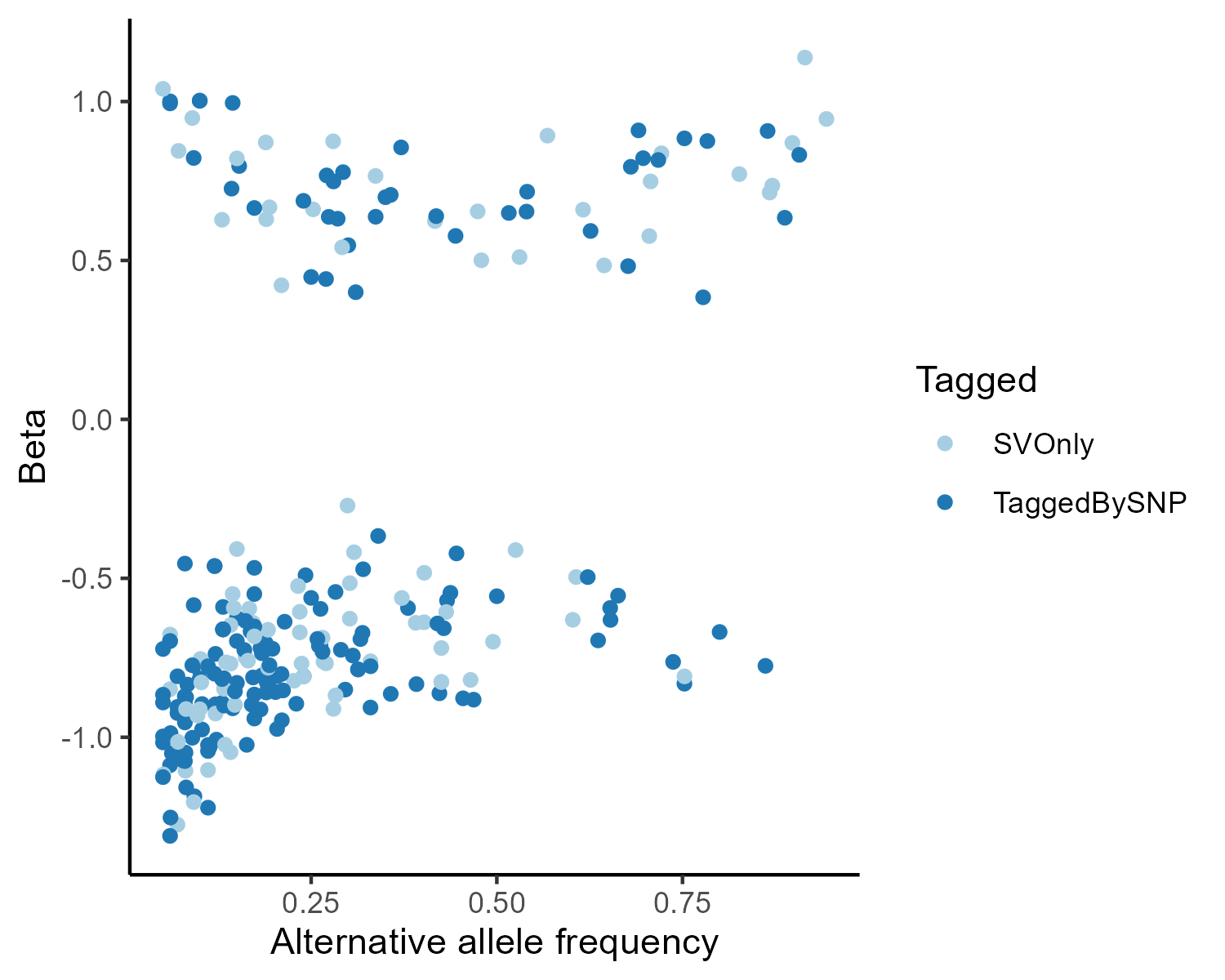


**Figure S10C**. Relationship between SV alternative allele frequency, effect size (Beta) and being tagged (in LD) with SNP.


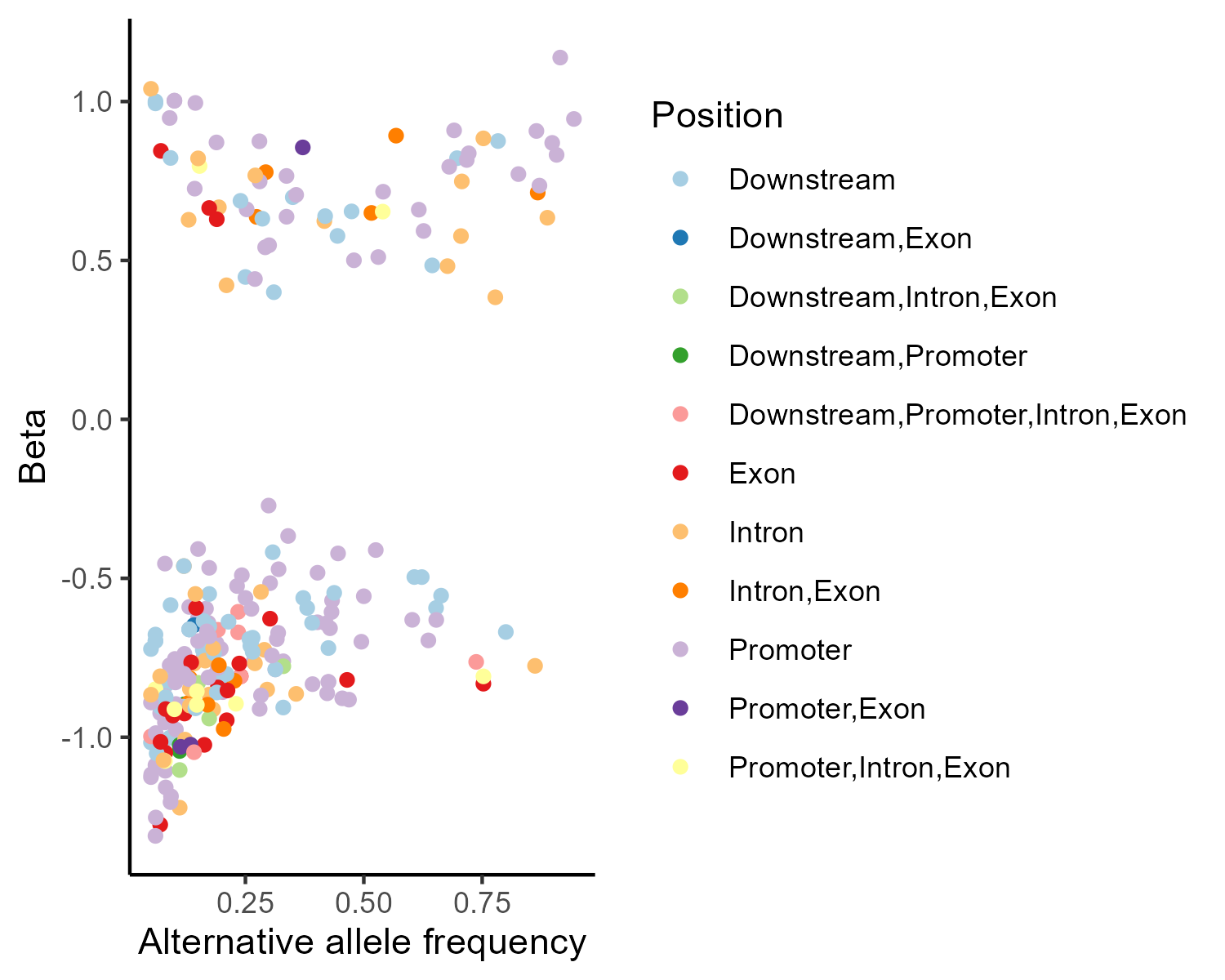


**Figure S10D**. Relationship between SV alternative allele frequency, effect size (Beta) and variant locations.


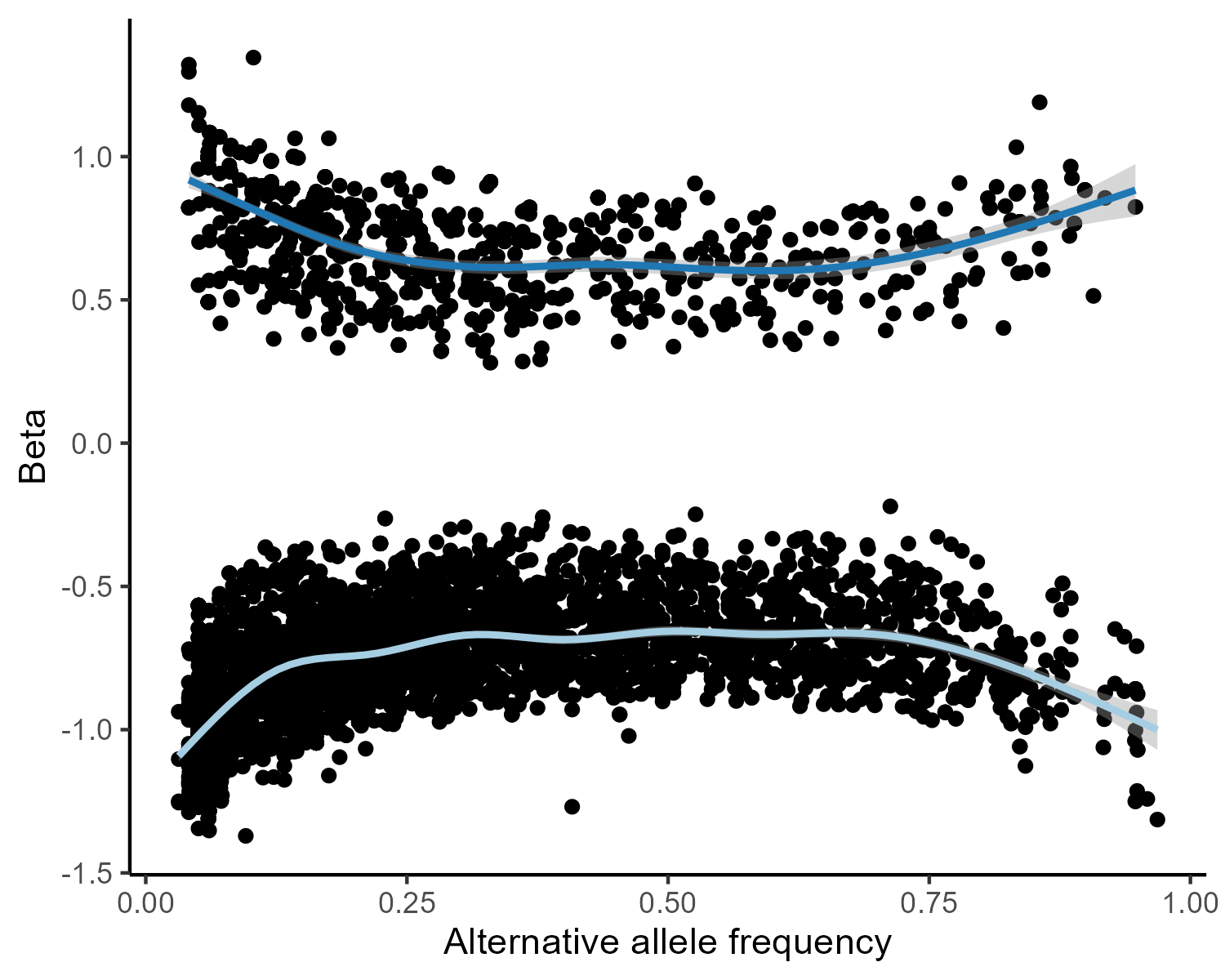


**Figure S11**. Relationship between SNP alternative allele frequency and effect size (Beta).


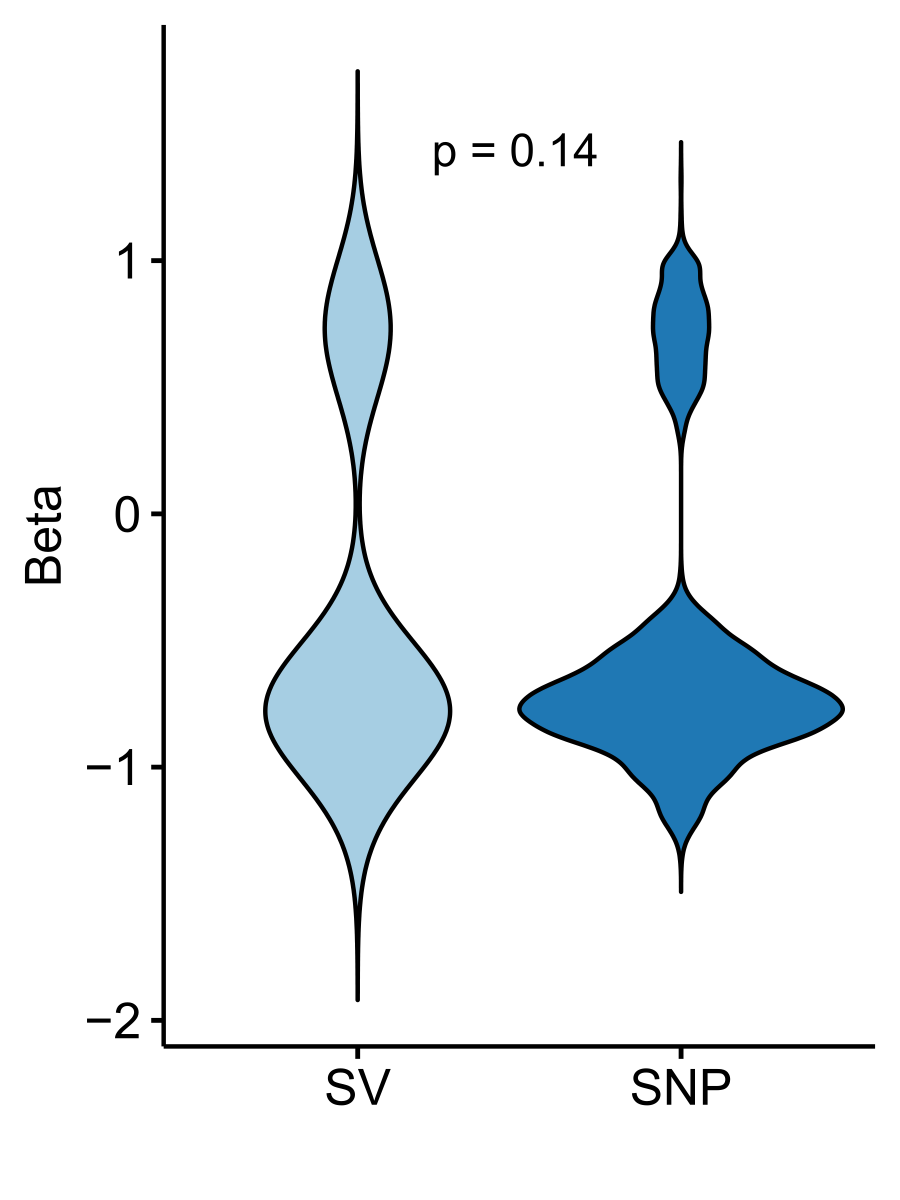


**Figure S12**. No difference in effect size between eQTL-SNPs and SVs was observed (Wilcoxon test, P=0.14).


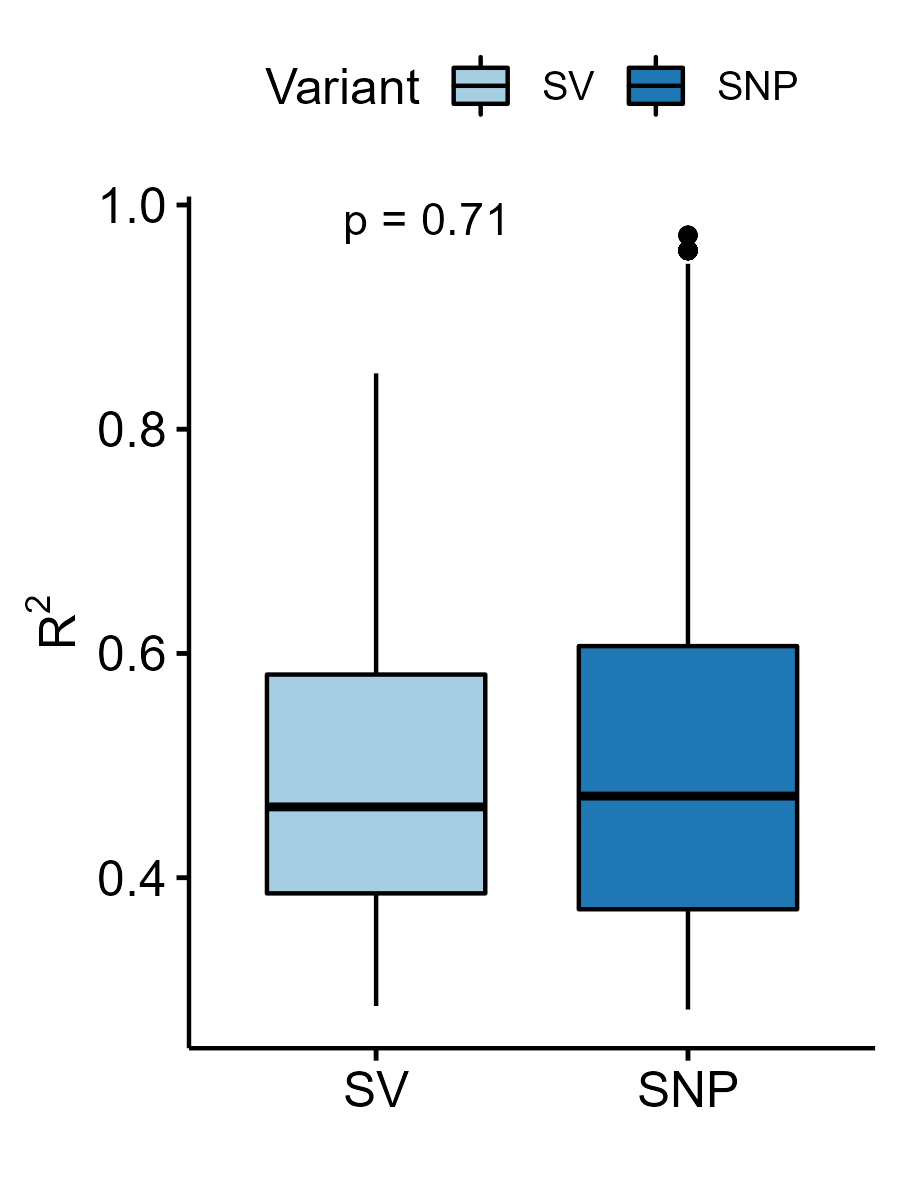


**Figure S13**. No difference of variance explained between eQTL-SNPs and SVs was observed (Wilcoxon test, P=0.71).
